# Trends in In-Hospital Cardiac Arrest and Mortality Among Children With Cardiac Disease in the Intensive Care Unit: A Systematic Review and Meta-analysis

**DOI:** 10.1001/jamanetworkopen.2022.56178

**Published:** 2023-02-10

**Authors:** Francesca Sperotto, Marco Daverio, Angela Amigoni, Dario Gregori, Anna Dorste, Catherine Allan, Ravi R. Thiagarajan

**Affiliations:** 1 Department of Cardiology, Boston Children’s Hospital, Harvard Medical School, Boston, Massachusetts; 2 Pediatric Intensive Care Unit, Department of Women’s and Children’s Health, University of Padova, Padova, Italy; 3 Laboratories of Epidemiological Methods and Biostatistics, Department of Environmental Medicine and Public Health, University of Padova, Italy; 4 Boston Children’s Hospital Library, Boston Children’s Hospital, Boston, Massachusetts

## Abstract

**Importance:**

Data on trends in incidence and mortality for in-hospital cardiac arrest (IHCA) in children with cardiac disease in the intensive care unit (ICU) are lacking. Additionally, there is limited information on factors associated with IHCA and mortality in this population.

**Objective:**

To investigate incidence, trends, and factors associated with IHCA and mortality in children with cardiac disease in the ICU.

**Data Sources:**

A systematic review was conducted using PubMed, Web of Science, EMBASE, and CINAHL, from inception to September 2021.

**Study Selection:**

Observational studies on IHCA in pediatric ICU patients with cardiac disease were selected (age cutoffs in studies varied from age ≤18 y to age ≤21 y).

**Data Extraction and Synthesis:**

Quality of studies was assessed using the National Institutes of Health Quality Assessment Tools. Data on incidence, mortality, and factors associated with IHCA or mortality were extracted by 2 independent observers. Random-effects meta-analysis was used to compute pooled proportions and pooled ORs. Metaregression, adjusted for type of study and diagnostic category, was used to evaluate trends in incidence and mortality.

**Main Outcomes and Measures:**

Primary outcomes were incidence of IHCA and in-hospital mortality. Secondary outcomes were proportions of patients who underwent extracorporeal membrane oxygenation (ECMO) cardiopulmonary resuscitation (ECPR) and those who did not achieve return of spontaneous circulation (ROSC).

**Results:**

Of the 2574 studies identified, 25 were included in the systematic review (131 724 patients) and 18 in the meta-analysis. Five percent (95% CI, 4%-6%) of children with cardiac disease in the ICU experienced IHCA. The pooled in-hospital mortality among children who experienced IHCA was 51% (95% CI, 42%-59%). Thirty-nine percent (95% CI, 29%-51%) did not achieve ROSC; in centers with ECMO, 22% (95% CI, 14%-33%) underwent ECPR, whereas 22% (95% CI, 12%-38%) were unable to be resuscitated. Both incidence of IHCA and associated in-hospital mortality decreased significantly in the last 20 years (both *P* for trend < .001), whereas the proportion of patients not achieving ROSC did not significantly change (*P* for trend = .90). Neonatal age, prematurity, comorbidities, univentricular physiology, arrhythmias, prearrest mechanical ventilation or ECMO, and higher surgical complexity were associated with increased incidence of IHCA and mortality odds.

**Conclusions and Relevance:**

This systematic review and meta-analysis found that 5% of children with cardiac disease in the ICU experienced IHCA. Decreasing trends in IHCA incidence and mortality suggest that education on preventive interventions, use of ECMO, and post-arrest care may have been effective; however, there remains a crucial need for developing resuscitation strategies specific to children with cardiac disease.

## Key Points

**Question:**

What are the trends in the incidence of and mortality after in-hospital cardiac arrest in pediatric patients with cardiac disease?

**Findings:**

In this systematic review and meta-analysis that includes data of 131 724 children with cardiac disease admitted to the intensive care unit, the incidence of in-hospital cardiac arrest and associated in-hospital mortality significantly decreased over time. The proportion of patients who did not achieve return of spontaneous circulation did not significantly change.

**Meaning:**

These findings suggest that efforts in education and prevention have been effective; however, there remains a crucial need for developing resuscitation strategies specific to children with cardiac disease.

## Teaser

This systematic review and meta-analysis investigates incidence, trends, and factors associated with in-hospital cardiac arrest (IHCA) and mortality in children with cardiac disease in the intensive care unit (ICU).

## Introduction

Children with cardiac disease are at high risk for heart failure, arrhythmias, cardiac arrest (CA), and death. The reported incidence of in-hospital CA (IHCA) and mortality in this population varies among studies. The incidence of IHCA among patients with cardiac disease admitted to the pediatric intensive care unit (ICU) ranges from 2.6 to 10%,^1,2,3,4,5,6,7,8,9,10,11,12^ and up to 12.7% in patients with single ventricle (SV) during their postoperative period following the Norwood stage 1 palliation.^13^ Overall, mortality rate remains high, ranging from 30 to 65%.^1,2,7,8,11,12,14,15,16,17,18^

In the last decades, the scientific community has put tremendous effort into education and prevention of CA in pediatric patients with cardiac disease. Since 2010, the American Heart Association (AHA) officially recognized the pediatric patient with cardiac disease as a high-risk patient for CA in its resuscitation guidelines, and strongly supported the consideration of extracorporeal membrane oxygenation (ECMO) as part of the resuscitation protocol (extracorporeal cardiopulmonary resuscitation [ECPR]) when expertise is available.^19,20^ In 2018, the AHA released a scientific statement entirely dedicated to the resuscitation of the pediatric patient with cardiac disease.^14^

Although there has been considerable effort in gathering data on IHCA in pediatric patients with cardiac disease, studies on incidence and mortality trends, which can help evaluate effectiveness of education and interventions, are lacking. The purpose of this systematic review and meta-analysis was to collect existing data, compute meta-data on IHCA incidence and associated mortality, and analyze their trends over the course of the last 2 decades. We also aimed to collect data on factors associated with IHCA and mortality.

## Methods

Data collection and reporting followed the Meta-analysis of Observational Studies in Epidemiology (MOOSE) reporting guideline^21^ suggested by the Enhancing the Quality and Transparency of Health Research (EQUATOR) Network. This meta-analysis study is exempt from ethics approval as we collected and synthesized data published from previous clinical studies in which informed consent had already been either obtained by the clinical investigators or waived due to the retrospective nature of the study. This study was registered in the National Institute for Health-Research (NIHR) international prospective register of systematic reviews (PROSPERO ID CRD42020156247).

### Data Sources and Search Strategy

A systematic search of PubMed (MEDLINE, PubMed Central, NCBI-Bookshelf, Cochrane), Web of Science, Embase, and Cumulative Index to Nursing and Allied Health Literature (CINAHL) was conducted from inception to September 2021. The search strategy was developed with the help of an expert librarian (A.D.) using both keywords and controlled vocabulary terms around the fields of (1) cardiac arrest, (2) cardiopulmonary resuscitation, (3) heart disease, and (4) intensive care (eMethods 1 in Supplement 1). The reference lists of the selected articles were also screened for completeness.

### Review Process and Study Selection

Studies were screened by two independent investigators with expertise in pediatric cardiology and critical care (F.S., M.D.) at title and abstract level. The same reviewers performed the full-text review and data extraction. Disagreements were discussed with a third investigator (A.A.) until consensus was reached.

**Figure 1.**
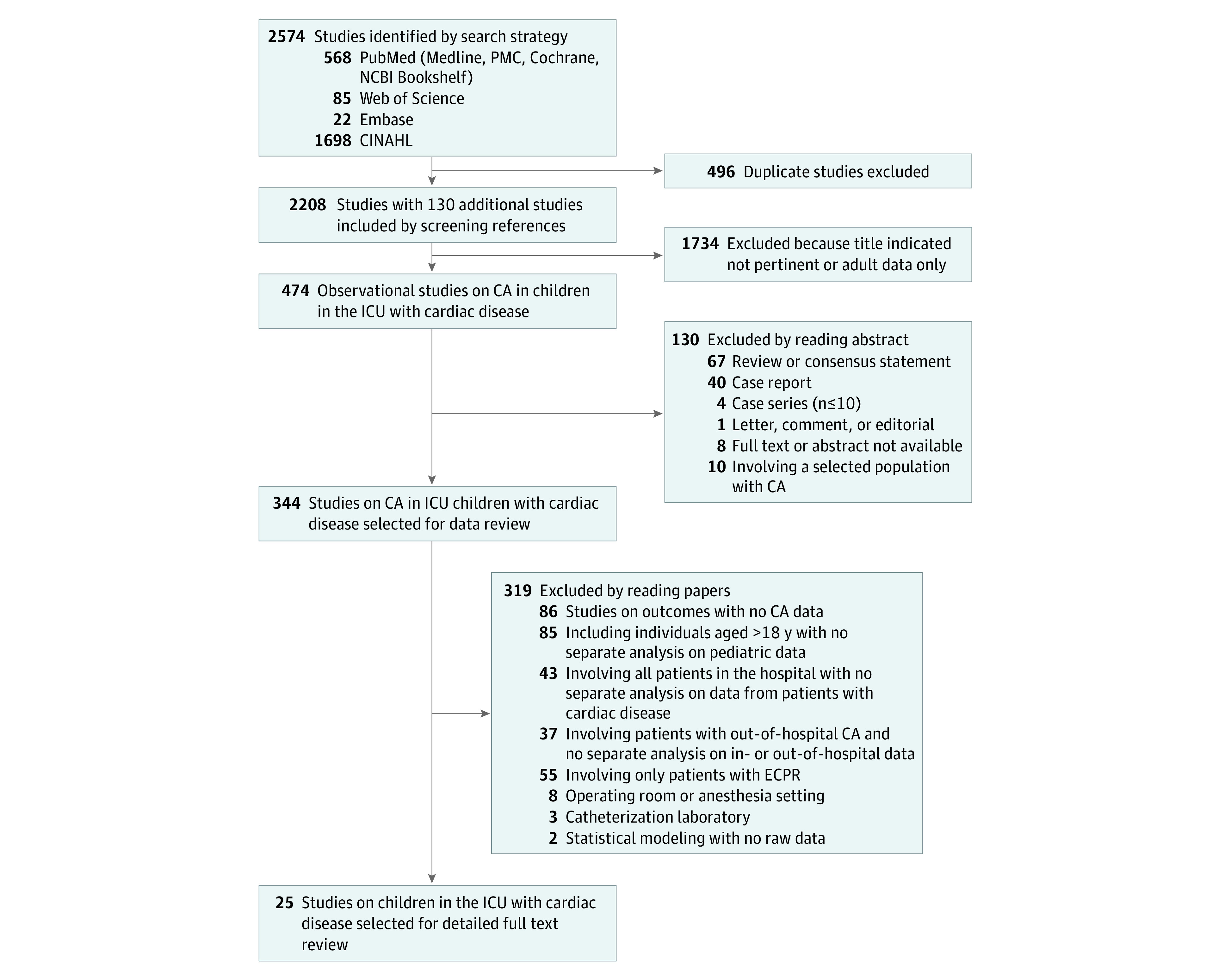
Flowchart of the Study Selection Process CA indicates cardiac arrest; ECPR, extracorporeal cardiopulmonary resuscitation; ICU, intensive care unit.

Studies were selected if they included data on cardiac patients admitted to a pediatric ICU at the time of the ICHA. Studies were excluded if they included a selected population (eg, IHCA during intubation only), had inseparable mixed populations of children vs adult (age cutoffs for children in the included studies varied from age less than or equal to 18 years to age less than or equal to 21 years), IHCA vs out-of-hospital CA, or general hospitalized patients vs ICU patients. Reviews, case reports, case series (n ≤10, for eliminating positive outcome bias), letters, and editorials were also excluded (Figure 1).

### Selection of Studies for Inclusion in the Meta-analysis, Quality Assessment, and Publication Bias

Selected studies were then evaluated for possible inclusion in the meta-analysis. Studies with duplicated data, those with a clear selection bias (eg, age limit), and conference abstracts were excluded. Selected studies at this level were analyzed for quality by 2 investigators (M.D., A.A.) independently using the National Heart Lung and Blood Institutes (NHLBI) Quality Assessment Tool for Observational Cohort or Case-Control Studies, as appropriate^22,23^ (eMethods 2 and eTable 1 in Supplement 1). Studies judged to be of poor quality based on the NHLBI tool were excluded from the meta-analysis. Publication bias was assessed using the funnel plot analysis.

### Data Extraction

Data were extracted by 2 independent investigators (F.S., M.D.). If multiple studies reported data from the same registry with overlapping timeframes, the study with the larger sample size was chosen for inclusion in the meta-analysis.

Primary outcome measures were incidence of IHCA and associated in-hospital mortality. Secondary outcomes were proportion of patients with absence of return of spontaneous circulation (ROSC) and proportion of those undergoing ECPR. Patients rescued with ECPR were considered as not achieving ROSC per definition. Additionally, we extracted data on odds ratios (ORs) of factors associated with IHCA and in-hospital mortality.

### Statistical Analysis

All statistical analyses were performed using R Statistics version 3.6.2 (R Project for Statistical Computing) from May to December 2022. Statistical significance was set at a 2-sided *P* < .05. Agreement on quality assessment was described as percentage and 95% CI using the binomial method and as a weighted *k*, with a range from 0.00 (no reproducibility) to 1.00 (perfect reproducibility).^24^ A random-effects meta-analysis using a logit transformation of the proportions and a generalized linear mixed effects modeling (GLMM) method was performed to compute the pooled incidence of IHCA, pooled proportion of patients not achieving ROSC, pooled proportion of patients undergoing ECPR, and pooled mortality rate. Some of the included studies were restricted to patients who experienced IHCA with no incidence data and were therefore not included in the incidence analysis. Pooled proportions were reported as percentages and 95% CI. Heterogeneity across studies was assessed using the *I*^2^ statistic. In case of considerable heterogeneity or publication bias, post hoc analyses were performed grouping studies by categories (time: last recruitment year before 2010 vs 2010 or later; type of study: registry-based vs nonregistry-based; category of patients: surgical vs general cardiac population). General cardiac population was defined as a mixed population of surgical and medical patients. Pooled ORs were computed by random-effects meta-analysis for factors evaluated by at least 2 studies based on crude ORs using the Mantel-Haenszel method and the DerSimonian-Laird and Paule-Mandel estimators. A random-effects metaregression model based on the restricted maximum likelihood estimator method was used to evaluate trends of IHCA incidence and mortality with last recruitment year as the main independent variable, adjusting for type of study and category of patients. Since type of study and time in the mortality model were collinear, this model was adjusted only for category of patients. The same analyses were performed using the mid recruitment year as a measure of time.

## Results

### Study Selection and Characteristics of the Included Study

A total of 2574 records were identified. Following removal of duplicates (n = 496), 2078 studies were screened on titles and abstracts. Of the remaining 344 full-text articles, 25 observational studies were included for the qualitative review, for a total of 131 724 children and 5213 IHCAs (4%)^25,26,27,28,29,30,31,32,33,34^ (Figure 1, Table). Of the 25 studies included, 23 were cohort studies and 2 were case-control studies. Twenty-two (88%) were retrospective and 3 prospective; 8 (32%) were multicenter and 17 (68%) single-center. Among the multicenter studies, 7 were registry-based. Twenty studies (80%) collected data from the US, the other 5 from Canada, Australia, United Kingdom, Finland, and Iran.

**Table.** Details of the Studies Selected for Inclusion

Source	Study design, setting, and study period	Patients	Sample size, No.	Age	Exclusion criteria	Definition of cardiac arrest	No. (%)		Outcome measures, No. (%)		Risk factors	
							Patients with CA	E-CPR	Short-term mortality	Late mortality	Cardiac arrest	Cardiac arrest associated mortality
**Studies including data on IHCA incidence**												
Dagan et al,^12^ 2019	Retrospective, single-center (Melbourne, Australia), 2007-2016	P-CICU patients post cardiac surgery	4983 admission (3781 patients)	Median 6 mo (IQR, 1-50 mo)	Children with medical cardiac conditions, children who had CA following procedures as cardiac catheterization, CA prior to cardiac surgery, DNR	Cessation of cardiac mechanical activity requiring cardiac massage for ≥1 min	211 (4.3)	NA	At discharge 64 (30.1)	NA	NA	Univariate analysis: younger age, male gender, lower weight, prematurity, chromosomal/genetic syndrome, need for ECMO/VAD, higher RACHS-1 category
Dhillon et al,^17^ 2018	Retrospective, single-center (Texas, USA), 2011-2016	P-CICU patients who experienced at least 1 CA	90 included (of 150 events over 5947 unique admissions)	NA	Multiple events in the same patient, events with incomplete documentation, CA outside the CICU	CPR ≥ 2 min	90 (150/5947; 2.5%)	23 (25.6)	No-ROSC^a^ 41 (46.0) (18 deaths, 23 ECPR); at 24h 25 (27.8); at discharge 49 (54.4)	NA	NA	Univariate analysis: no epinephrine infusion pre-CA, no arterial line pre-CA (surgical patients), longer CA duration (surgical patients), higher number of epinephrine doses (surgical patients)
Alten et al,^11^ 2017	Retrospective analysis of prospective data, PC4 registry, multicenter (23 USA centers), 2014-2016	P-CICU patients (medical and surgical)	15 908^b^	Range 0-18 y	None	Cardiopulmonary arrest requiring chest compressions and/or defibrillation for pulseless VT or acute respiratory compromise requiring emergency assisted ventilation leading to cardiopulmonary arrest requiring chest compressions and/or defibrillation	492 CICU encounters (3.1)	134 (27.2)	No-ROSC^a^ 174 (35.5) (40 deaths, 134 ECPR); at 24h 129 (26.2); at discharge 230 (46.7)	NA	Multivariable model: surgical patients: premature neonate, term neonate, infant, underweight, any chromosomal abnormality/syndrome, any STS preop. risk factor, STS-EACTSCHS mortality category 4 or 5; medical patients: premature neonate, medical condition, acute HF, lactate>3 mmol/L within 2 h of CICU admission, MV 1hr post CICU admission	NA
Gupta et al,^10^ 2016	Retrospective analysis of prospective data, VPS (NACHRI) registry, multicenter (62 USA centers) 2009-2014	P-CICU patients with CHD post cardiac surgery	26 909	Mean (SD) 37.6 mo (55.7)	ICU readmission, lack of surgical documentation, surgical closure of isolated PDA or surgery not listed in STS-EACTS	Any event characterized by either pulselessness or critically compromised perfusion treated with external chest compression and/or defibrillation	736 (2.7)	NA	At discharge 229 (31.1)	NA	Multivariable model; risk factors: younger age, female gender, development disorder, high complexity operations, MV before surgery, higher PIM-2 score, SV anatomy, PH, acute lung injury, RI, chylothorax, arrhythmia, seizures, brain hemorrhage, MV after surgery, hemodialysis catheter; protective factors: younger age (>28 d, <1 y), higher weight, arterial line, attending intensivist.	Multivariable model; risk factors: ECMO, SV anatomy, RI, brain hemorrhage, hemodialysis catheter; protective factors: younger age (<28 d), presence of cardiac PICU.
McMillan et al,^25^ 2016^c^	Retrospective, single-center (Baltimore, USA)	P-CICU patients post cardiac surgery	461	<21 y	NA	NA	27 patients (5.9), 34 events	5/27 (18.5)	No-ROSC^a^ 7/27 (2 deaths, 5 ECPR) (25.9); at discharge 9/27 (33.0)	NA	NA	NA
Butts et al,^9^ 2014	Retrospective analysis of prospective randomized trial, single-center (Charleston, USA) 2007-2009	Neonates post cardiac surgery with CPB	76	Range 0-1 mo	<36 wk gestational age at time of surgery, previous treatment or contraindication to steroid therapy, preoperative use of mechanical circulatory support or active resuscitation at time of proposed randomization	CPR as CA requiring chest compression	3 (3.9)	0 (0.0)	No-ROSC 0 (0.0)	NA	NA	NA
Kalfa et al,^26^ 2014^d^	Retrospective, single-center (New York, USA), 2006-2012	P-CICU Neonates with CHD and weight<2.5 kg post cardiac surgery	146^b^	Mean (SD) 18.2 (24.2)	Patients who underwent isolated PDA closure	Not defined	18 (12.3)	NA	At discharge 14 (77.8)	NA	NA	NA
Gupta et al,^8^ 2014	Retrospective analysis of prospective data, STS-CHSD registry, multicenter (97 USA centers), 2007-2012	P-CICU patients with CHD post cardiac surgery	70,270	Median 156 d (IQR, 21-1359 d)	Surgery not classified into one of the STS-EACTS mortality categories, missing outcome data	Cessation of effective cardiac mechanical function	1843 (2.6)	NA	At discharge 910 (49.4)	NA	Univariate model; female sex, lower age, lower weight, prematurity, congenital disorders, preop. LOS, preop. MV, preop. sepsis, preop. shock, preop. RI, preop. CPR, CPB time, previous cardiothoracic surgery, STS-EACTS mortality high risk, STS morbidity high risk; multivariable model: NS	Multivariable model; low volume centers (<150 case/y), low-medium volume centers (150-250 case/y), STS-EACTS mortality risk category 1-3 in low and in medium volume centers,; STS-EACTS mortality risk category 4-5 in low and medium-low volume centers.
Ahmadi et al,^27^ 2013^c^	Single-center (Tehran, Iran), 2001-2002	P-CICU patients <7 years of age, post cardiac surgery	529	<7 y	Not defined	Not defined	59 (11.2)	NA	At discharge 37 (62.7)	NA	NA	Univariate analysis; lower mean arterial BP before the CA
Watkins et al,^28^ 2013^c^	Retrospective, STS registry, single-center (Nashville, USA) 2006-2011	P-CICU patients with CHD and RACHS1 category 6 undergoing surgical non cardiac procedures	71	Range, 0-18 y	Not defined	Requirement for chest compressions, electrical defibrillation or cardioversion, or initiation of pharmacotherapy	3 (4.2)	2 (66.7)	No-ROSC^a^ 2 (66.6) (0 death, 2 ECPR); at 24h 0 (0.0)	NA	NA	NA
Argawal et al,^29^ 2012	Prospective, single-center (Nashville, USA), 2007-2010	P-CICU patients post cardiac surgery	1078	Range, 0-18 y	Patients managed in NICU, adult ICU and pediatric cardiology floor	Not defined	48 (4.5)	NA	NA	NA	NA	NA
Gaies et al,^7^ 2012	Retrospective, single-center (Ann Arbor, USA) 2006-2008	P-CICU patients with at least 1 episode of CA	102 (of 2230 P-CICU admission)	Median 79 d (IQR, 12-420)	Not defined	Event requiring active chest compressions for any duration	102 (4.6)	10 (9.8)	No-ROSC ^a^ 27 (16.5) (17 death, 10 ECPR); at discharge 53 (52.0)	NA	NA	Multivariable model: arrest during weekend, experience of primary nurse <1y, VIS≥20
Hansen et al,^30^ 2011	Case-control, single-center (Edmonton, Canada) 1996-2005	NICU patients post cardiac surgery with CPB, ≤6 weeks of age. Cases: with at least 1 CPR event, controls: without CPR events	29 CA (cases) (of 343 patients post cardiac surgery)	NA	Cardiac surgery not requiring CPB, patients having CPR preoperatively or in the operating room	Not defined	CPR 29 (8.5)	9 (31.0)	No-ROSC ^a^ 17 (8 death, 9 ECPR) (58.6)	At 1 month 11 (37.9);at 2 y 17 (58.6)	Univariate analysis: lower birth weight, and gestational age, longer preoperative ventilator days, and worse postoperative day 1 peak lactate, base deficit, pH, and inotrope score	Multivariable model on all cohort, not on patients with CA only: minutes of chest compression
Ades et al,^31^ 2010^c^	Retrospective, single-center (Philadelphia, USA), 2000-2004	Patients with CHD and low birth weight (<2.5 kg) post cardiac surgery	105	Median 5 d, range 0-125 d	Patients who underwent isolated PDA closure	Not defined	23 (21.9)	Not ECPR Center	No-ROSC 7 (30.4); at discharge 17 (73.9)	NA	NA	NA
Gaies et al,^6^ 2010^c^	Retrospective, single-center (Ann Arbor, USA), 2007-2008 overlap data	P-CICU patients post cardiac surgery with CPB	173	Range, 0-6 mo	Not defined	Not defined	15 (8.7)	NA	NA	NA	NA	NA
Dorfman et al,^5^ 2008	Retrospective, single-center (Philadelphia, USA), 2002-2003	P-CICU and NICU neonates with cardiac disease	190	Median 1 d (range, 0-27)	Neonates with recovery from anesthesia or sedation from a non-cardiac procedure; <37 weeks’ gestation admitted to NICU with an isolated PDA or PFO, asymptomatic ASD or VSD transferred to the NICU for a specific non-CV pediatric subspecialty evaluation	Not defined	CPR 18 (9.5)	NA	NA	NA	NA	NA
Gillespie et al,^32^ 2006	Retrospective, single-center (Philadelphia, USA), 2000	P-CICU patients with CHD and <6months post cardiac surgery	221	Range, 0-6 mo	Not defined	Not defined	19 (8.7)	NA	NA	NA	NA	NA
Brown et al,^4^ 2003	Retrospective, single-center (London, UK), 1999-2000	P-CICU patients post cardiac surgery with CPB	342	Range, 0-18 y	Incomplete data, unclassifiable operation, multiple admissions	Not defined	CPR 34 (9.9)	NA	NA	NA	NA	NA
Suominen et al,^3^ 2001	Case control; single-center (Helsinki, Finland), 1990-1994	P-CICU patients with CHD post cardiac surgery. Cases: with at least 1 episodes of CA, Controls1: with DHCA without CA, Controls2: without DHCA without CA	82 CA (48 CPR, 44 resuscitation not attempted) (of 1,115 post cardiac surgery patients)	Range, 0-18 y	Patients who only received resuscitation drugs or MV, or who had received CPR in the operating theater	Absence of consciousness, apnea, and lack of palpable pulses in major arteries	82 (7.3); CPR 48 (44 resuscitation not attempted)	Not ECPR Center	No-ROSC: 21/48 (43.8); at discharge: 39/48 (81.3)	At 1-y: 39/48 (81.2)	Univariate analysis: younger age, SV physiology, preop. MV, PGE1, preop. inotropic support, longer mean aortic-cross-clamp time, longer CPB time, longer DHCA time, higher inotropic support during surgery, and higher postop. inotropic support	NA
Rhodes et al,^1^ 1999	Retrospective, single-center (New York, USA), 1994-1998	P-CICU patients with CHD and age <12months post-cardiac surgery	575	Range, 0-12 mo	Not defined	Chest compressions or the absence of a palpable spontaneous pulse that was not resolved with only airway intervention	34 (5.9)	Not ECPR Center	No-ROSC 11 (32.4); at discharge: 20 (58.8)	At 6 mo 20 (58.8); at follow-up (median 21 mo) 21 (61.8)	NA	Univariate analysis; lower pre-arrest MAP, lower arterial pH, higher epinephrine doses, higher bicarbonate dose, longer CPR duration
**Studies focusing on a cohort of patients who experienced IHCA, with no IHCA incidence data included**												
Perry et al,^33^ 2020 (Conference abstract)^c^	Retrospective, multicenter, GWTG-R registry, 2014-2018	PICU or P-CICU patients (medical and surgical) who underwent CPR	866	<18 y	NA	NA	866	162 (18.7)	No ROSC^a^ 283 (32.7) (121 deaths, 162 ECPR); at discharge 364 (42.0)	NA	NA	NA
Yates et al,^16^ 2019	Prospective, multicenter (PICqCPR study, USA centers, CPCCRN network), 2013-2016	PICU or P-CICU patients (medical and surgical) with invasive arterial blood pressure monitoring line prior and during CPR	113	Range, 0-19 y	Patients for which first compression was not captured on the waveform data, or compression start and stop could not be determined	CPR for at least 1 min	113	33 (29.2)	No ROSC^a^ 72 (63.7) (39 deaths, 33 ECPR); at discharge 56 (49.6)	NA	NA	Univariate analysis: diastolic BP ≥25 mmHg for infants or ≥30 mmHg for children (surgical patients)
Berg et al,^15^ 2016	Prospective, CPCCRN registry, multicenter (6 USA centers), 2011-2013	Cardiac patients cohort of PICU patients with at least 1 episode of CA	73	Range, 0-18 y	Patients with vital signs incompatible with life for at least the first 2 h after PICU admission (ie, moribund patients)	CPR event: chest compressions for >1 min and /or defibrillation. The reasons for initiation of chest compressions were categorized as a pulseless CA or poor perfusion with bradycardia and/ or hypotension	73	NA	No-ROSC^a^ 16 (21.9); at discharge 41 (56.2)	NA	NA	NA
Gupta et al,^34^ 2014 PCCM	Retrospective, Multicenter (3 USA Centers) 2002-2010	Cardiac patients cohort of PICU patients with at least 1 episode of CA	170	NA	Out-of-hospital, out-of-PICU, CPR ongoing at admission, patients receiving only drugs and/or MV without chest compressions and/or defibrillation	Monitored cardiopulmonary arrest treated with chest compressions for >1 min	170	NA	No-ROSC^a^ 35 (20.6); at 24h 48 (28.2); at discharge 91 (53.5)	NA	NA	NA
Parra et al,^2^ 2000	Retrospective, single-center (Miami, USA), 1995-1997	P-CICU patients with at least 1 episode of CA	32	Median, 1 mo (range, 1 d- 21 y)	DNR patients	Cessation of circulation and respiration that required CPR for>2 mins	32 (38 events)	4 (12.5)	No-ROSC^a^ 18 (56.2) (14 deaths, 4 ECPR); at discharge: 18 (56.2)	At 6 mo: 21 (65.6)	NA	Univariate analysis: NS

Abbreviations: CA, cardiac arrest; CHD, congenital heart disease; CPB, cardiopulmonary bypass; CPR, cardiopulmonary resuscitation; CV, cardiovascular; DHCA, deep hypothermic circulatory arrest; DNR, do not resuscitate; E-CPR, ECMO-cardiopulmonary resuscitation; HF, heart failure; MAP, mean arterial pressure; MV, mechanical ventilation; NA, not available; NICU, neonatal intensive care unit; NS, not significant; P-CICU, pediatric cardiac intensive care unit; PH, pulmonary hypertension; PICU, pediatric intensive care unit; preop., preoperative; postop., postoperative; RACHS-1, risk adjustment for congenital heart surgery-1; RI, renal insufficiency; ROSC, return of spontaneous circulation; SV, single-ventricle; VAD, ventricular assist device.

^a^ For the purpose of this meta-analysis, no-ROSC is defined as absence of a spontaneous return of circulation, thus it also includes patients who needed ECMO to restore circulation.

^b^ The study included 1197 patients aged at least 18 years, however, since those represent only the 7.5% of the total sample, the study was included.

^c^ Excluded from the meta-analysis. Reasons for exclusion were: Perry 2020 and McMillan 2016: conference abstracts; Gaies 2010: duplicate data with Gaies 2012; Kalfa 2014 and Ades 2010: including patients with cardiac disease greater than 2.5 kg and less than 4 kg only, respectively; Watkins 2013: including cardiac patients belonging to the RACHS-1 category 6 only.

^d^ The study included 768 children with cardiac disease less than 4 kg, however data on CA incidence and associated mortality are available only for the cohort less than 2.5 kg, N = 146.

### Selection of Studies for Inclusion in the Meta-analysis and Quality Assessment

Of the 25 studies identified for the qualitative review, 19 were judged to be candidates for inclusion in the meta-analysis: we excluded 2 conference abstracts, one study with duplicate data, and 3 studies with biased populations (Table). The 19 studies identified were evaluated for quality (eTable 1 in Supplement 1). Most of the studies were judged to be of fair or good quality (18 of 19 [95%]). Reviewers’ quality classification was concordant in 16 of 19 studies (84% [95% CI, 62-95%] agreement) for a weighted *k* statistic of 0.76. Because one study was judged to be of poor quality based on the NHBLI tool, 18 studies were ultimately included in the meta-analysis (129 373 patients and 4195 [3%] IHCAs).

### Incidence Rate of IHCA and Trend Over Time

**Figure 2.**
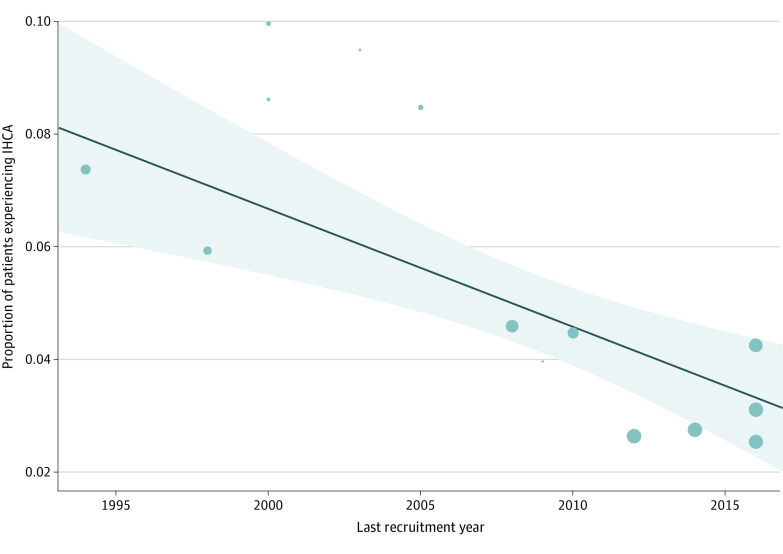
Trend in Incidence of In-Hospital Cardiac Arrest (IHCA) Over Time by Metaregression The incidence of IHCA in pediatric patients with cardiac disease in the intensive care unit significantly decreased in the last 20 years (*P *for trend < .001). The model was adjusted for type of study (registry-based vs nonregistry-based) and diagnostic category (surgical vs general cardiac).

By random-effects meta-analysis, a pooled proportion of 5% (95% CI, 4%-6%) of pediatric patients with cardiac disease experienced IHCA in the ICU (eFigure 1 in Supplement 1). Post hoc analyses according to time and type of study were able to reduce heterogeneity while controlling the publication bias (eFigures 2-4 in Supplement 1). The pooled proportion of patients experiencing IHCA in studies with last recruitment year before 2010 was 7% (95% CI, 5%-8%), whereas last recruitment year of 2010 or later was 3% (95% CI, 2%-4%). The incidence rate of IHCA was higher among nonregistry-based studies (6% [95% CI, 4%-8%]) compared with registry-based studies (3% [95% CI, 2%-3%]). The post hoc analysis based on patient categories was not able to reduce heterogeneity, and incidence was similar in the 2 categories (surgical: 5% [95% CI, 4%-7%]; general cardiac population: 4% [95% CI, 2%-8%]). By random-effects metaregression adjusting for study type and category of patients we found that the incidence rate of IHCA significantly decreased over time (*P* for trend < .001) (Figure 2); additionally, this was confirmed when using the mid recruitment year as a measure of time (*P* for trend < .001) (eFigure 5 in Supplement 1).

### Use of ECMO, ROSC, Mortality Rate, and Trends Over Time

**Figure 3.**
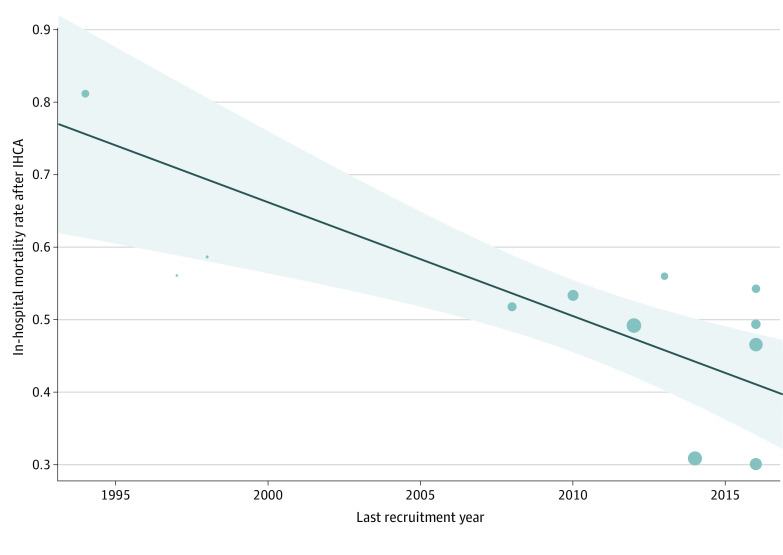
Trend in In-Hospital Mortality After In-Hospital Cardiac Arrest (IHCA) Over Time by Metaregression The in-hospital mortality rate after IHCA in pediatric patients with cardiac disease in the intensive care unit significantly decreased in the last 20 years (*P* for trend < .001). The model was adjusted for diagnostic category (surgical vs general cardiac).

Overall, 39% (95% CI, 29%-51%) of patients did not achieve ROSC (eFigures 6 in Supplement 1). In ECMO centers, 22% (95% CI, 14%-33%) underwent ECPR, while 22% (95% CI, 12%-38%) were unable to be resuscitated (eFigures 7-8 in Supplement 1). The overall pooled in-hospital mortality was 51% (95% CI, 42%-59%) (eFigure 9 in Supplement 1). Post hoc analyses according to time, type of study, and category of patients showed improved heterogeneity and decreased publication bias (eFigures 10-12 in Supplement 1). For studies with last recruitment year prior to 2010, the pooled in-hospital mortality was 62% (95% CI, 41%-80%), whereas for studies with last recruitment year of 2010 or later was 46% (95% CI, 37%-54%). Within nonregistry-based studies, mortality was higher than reported by registries (54%, [95% CI, 42%-65%] vs 45% [95% CI, 31%-60%]). The in-hospital mortality in the surgical and general cardiac cohorts were similar (55% [95% CI, 25%-82%] vs 49% [95% CI, 41%-56%]). By random-effects metaregression adjusting for study type and category of patient*s*, we found that the proportion of patients who did not achieve ROSC did not significantly change over time (*P* for trend = .90), whereas in-hospital mortality after IHCA significantly decreased (*P* for trend < .001) (Figure 3). These results were supported when the mid recruitment year was used as a measure of time (eFigure 13 in Supplement 1).

### Factors Associated With IHCA and Mortality

By random-effects meta-analysis, factors found to be associated with IHCA were neonatal age, genetic syndrome, univentricular physiology, arrhythmias, pulmonary hypertension, kidney failure, sepsis, seizures, mechanical ventilation, prearrest ECMO support, recent surgery with circulatory arrest, and higher surgical complexity (Society of Thoracic Surgeons [STS] category 4-5; eTable 2 in Supplement 1). The main pooled factor associated with in-hospital mortality after IHCA was higher surgical complexity (STS category 4-5; eTable 3 in Supplement 1).

## Discussion

This systematic review and meta-analysis on the incidence, mortality, and risk factors for IHCA in pediatric ICU patients with cardiac disease brings together data from 25 studies, for a total of 131 724 patients. By proportion meta-analysis, we found that 5% of pediatric ICU patients with cardiac disease experienced IHCA. Notably, by adjusted metaregression, we found that the incidence of IHCA significantly decreased in the last 2 decades. Among patients who experienced IHCA, a substantial proportion (39%) did not achieve ROSC. ECMO has become a crucial part of the resuscitation strategy, with approximately 22% of patients with IHCA undergoing ECPR. In-hospital mortality unfortunately remains high at 51%, but significantly decreased over time. However, the proportion of patients who did not achieve ROSC did not significantly change.

Hospitalized children with cardiac disease are at increased risk for CA.^14,35^ Most children with cardiac disease have a congenital heart defect (CHD), in which circulatory pathways may be abnormal. These patients require either surgical correction or palliation and may therefore have residual lesions which impact cardiac physiology, or may develop new structural or functional lesions over time. They are also at higher risk of myocardial dysfunction, arrhythmias, and—in the case of patients with SV—unbalanced systemic and pulmonary circulations. In these patients, cardiorespiratory interactions have a greater impact on hemodynamics. Finally, the anatomic and physiologic substrates of CHDs influence the effectiveness of the resuscitation strategy, especially in neonates and patients with SV.^14^

In this study, we found that the incidence of IHCA among pediatric ICU patients with cardiac disease significantly decreased over the course of the last 2 decades. During this time frame, the scientific community has put substantial effort into developing specific guidelines targeted to the pediatric population with cardiac disease,^14,19,20^ with particular focus on prevention, use of ECPR, and optimization of postarrest care.

Resuscitation may be particularly challenging in patients with cardiac disease, thus substantial attention should be made in preventive measures.^14^ For example, maintenance of effective CPR may be more difficult in patients with parallel circulation, severe atrioventricular or semilunar valvar regurgitation, or in those with passive pulmonary blood flow such as the bidirectional cavopulmonary anastomosis or Fontan circulations. In patients with SV physiology and parallel circulations, chest compression may drive blood flow substantially more in the circulation with lower resistance (usually pulmonary) with consequent significant systemic hypoperfusion.^14^ In patients with cavopulmonary anastomosis or Fontan circulations, pulmonary blood flow is passive, depends on systemic venous preload, and is affected by pulmonary vascular resistance (PVR); in the setting of acidosis, PVR increases limiting the pulmonary blood flow, decreasing the cardiac preload and impairing the ability to generate effective cardiac output.^36^ Additionally, cardiac patients often have different degrees of heart failure secondary to pressure or volume overload conditions, previous ischemic event, restrictive physiology, or baseline myocardial disease, which lead to reduced myocardial reserve, higher likelihood of systemic hypoperfusion and associated acidosis, and ineffective myocardial response to standard resuscitation efforts. The AHA guidelines focus on specific recommendations for each cardiac physiology to help optimize patient care and reduce the risk of IHCA. For example, oxygen administration, permissive hypercarbia, vasodilators and vasopressors may be used to correct imbalances between the pulmonary and systemic circulations (Qp/Qs) in patients with parallel circulations.^14,19,20^ Also, since occlusion of the shunt—often the sole source of pulmonary blood flow—may rapidly cause cardiovascular collapse, anticoagulation may be considered.^14,37^ Finally, patients with cavopulmonary anastomosis or Fontan physiology in the prearrest phase may benefit from afterload reduction or gentle positive pressure ventilation to reduce systemic ventricular afterload while avoiding excessive mean airway pressures that may impair pulmonary blood flow.^14,38,39^ Notably, recent quality improvement studies demonstrated the efficacy of specific preventive measures in reducing the incidence of IHCA in this high-risk population.^40,41^

The AHA guidelines also emphasize the use of ECPR in centers with expertise. ECMO allows a nonspontaneous return to circulation with the opportunity to correct residual lesions, physiological imbalances, or to bridge to transplant or ventricular assist devices. Here, we found that a substantial proportion of patients (22%) underwent ECPR. An increased use of ECMO for ECPR over time was reported by the European Life Support Organization, with increased number of pediatric ECPR cases entered in the registry among both surgical and medical patients with cardiac disease.^42,43^ Our study first provides a pooled estimate of the proportion of pediatric IHCA patients who benefit from ECMO as a resuscitation strategy.

Our study also found that mortality after IHCA in pediatric cardiac ICU patients remains high. A pooled proportion of 39% of patients were not able to achieve ROSC, and half of the patients (51%) did not survive to hospital discharge. Interestingly, the proportion of patients who did not achieve ROSC did not significantly change over time, while in-hospital mortality significantly decreased. This may be because cardiopulmonary resuscitation techniques remained overall similar over time, while use of ECMO increased and postarrest care was improved. This illustrates the crucial need for developing resuscitation strategies specific to children with cardiac disease. For example, given that patients with SV have the highest risk of IHCA, researchers are investigating optimal CPR strategies suggesting an optimal chest compression position corresponding to the 25% of the lower sternum,^44^ or the use of interposed abdominal compression CPR in patients after the Norwood stage 1 palliation.^45^ Specific resuscitation strategies—with respect to chest compressions, ventilation, and pharmacologic approach—should be investigated for children with different physiologies, such as children with parallel vs in-series circulation or in those with passive vs antegrade pulmonary blood flow. Studies are also needed to investigate the role of specific drugs for which the level of evidence is low, such as calcium and bicarbonates.^14^ Reassuringly, the in-hospital mortality rate over time significantly decreased, suggesting that education on postarrest care—and use of ECMO—have improved outcomes in this high-risk population.

### Limitations

This study has several limitations. First, this is a meta-analysis of observational studies with high and difficult-to-control heterogeneity of data, which may decrease the statistical certainty of the findings. Second, there are no data regarding the actual compliance to the AHA guidelines; however, given the worldwide diffusion of the AHA Pediatric Advanced Life Support training and given that 80% of the studies were performed in the US, it seems reasonable to assume that medical practice has been influenced by those recommendations. This assumption has been previously used in studies evaluating the impact of guidelines in other contexts, such as endocarditis.^46^ Third, the definition of CA varies among studies; however, we believe that each of these events may be considered as an acute cardiovascular collapse requiring CPR, and may be pooled together. Fourth, although we adjusted for major confounders, there is still a risk of confounding by age, baseline physiology, resuscitation features such as CPR quality (chest compression rate, depth, recoil), hemodynamic data, resuscitation resources, and postarrest care; also, given the small number of studies comparing medical vs surgical patients, we were not able to compare these 2 categories specifically, but only the surgical vs general cardiac ICU patients. Fifth, data on outcomes of ECPR patients are limited because this was not the main research question; future studies focused on ECPR will be able to pool more informative data. Additionally, we did not focus on neurologic or functional outcomes.

## Conclusions

This systematic review and meta-analysis found that a nonnegligible proportion (5%) of children with cardiac disease in the ICU experienced IHCA. ECMO has become an important resuscitation strategy in this high-risk population, with about one-quarter of patients undergoing ECPR in centers with expertise. Both the incidence of IHCA and associated in-hospital mortality have significantly decreased over time, suggesting that education on preventive measures, the use of ECMO, and postarrest care, may have significantly improved outcomes. However, the unchanged proportion of patients not achieving ROSC illustrates the crucial need to develop resuscitation strategies specific to children with cardiac disease.

## Notes

Supplement 1. **eMethods 1.** Detailed Search Strategy **eMethods 2.** National Heart, Lung, and Blood Institute (NHLBI) Quality Assessment Tool for Observational Studies Checklist **eMethods 3.** National Heart, Lung, and Blood Institutes (NHLBI) Quality Assessment Tool for Case-Controls Studies Checklist **eTable 1.** Quality Assessment of Candidate Studies for the Meta-Analysis **eTable 2.** Pooled Odd Ratios for Risk Factors For In-Hospital Cardiac Arrest (Factors Included in at Least Two Studies) **eTable 3.** Pooled Odd Ratios for Risk Factors for In-Hospital Mortality After In-Hospital Cardiac Arrest (Factors Included in at Least Two Studies) **eFigure 1.** Pooled Proportion of Patients Experiencing In-Hospital Cardiac Arrest by Random Effects Meta-Analysis **eFigure 2.** Pooled Proportion of Patients Experiencing In-Hospital Cardiac Arrest by Random Effects Meta-Analysis According to Last Recruitment Year (<2010, Upper, and ≥2010, Lower) **eFigure 3.** Pooled Proportion of Patients Experiencing In-Hospital Cardiac Arrest by Random Effects Meta-Analysis in Non-Registry-Based Study (Upper) and in Registry-Based Studies (Lower) **eFigure 4.** Pooled Proportion of Patients Experiencing In-Hospital Cardiac Arrest by Random Effects Meta-Analysis According to the Category of Patients (Surgical, Upper, and General Cardiac, Lower) **eFigure 5.** Trend in Incidence of In-Hospital Cardiac Arrest Over Time by Meta-Regression Using Mid-Recruitment Year as a Measure of Time **eFigure 6.** Pooled Proportion of Patients Who Did Not Achieve Return of Spontaneous Circulation by Random Effects Meta-Analysis **eFigure 7.** Pooled Proportion of Patients Undergoing Extracorporeal Cardiopulmonary Resuscitation by Random Effects Meta-Analysis **eFigure 8.** Pooled Proportion of Patients Who Died Suddenly in Centers With Extracorporeal Cardiopulmonary Resuscitation Expertise, by Random Effects Meta-Analysis **eFigure 9.** In-Hospital Pooled Mortality Rate for In-Hospital Cardiac Arrest by Random Effects Meta-Analysis **eFigure 10.** In-Hospital Pooled Mortality Rate for In-Hospital Cardiac Arrest by Random Effects Meta-Analysis According to Last Recruitment Year (<2010, Upper, and ≥2010, Lower) **eFigure 11.** In-Hospital Pooled Mortality Rate by Random Effects Meta-Analysis in Non-Registry-Based Studies (Upper) and in Registry-Based Studies (Lower) **eFigure 12.** In-Hospital Pooled Mortality Rate for In-Hospital Cardiac Arrest by Random Effects Meta-Analysis According to Category of Patients (Surgical, Upper, and General Cardiac, Lower) **eFigure 13.** Trend in In-Hospital Mortality After In-Hospital Cardiac Arrest Over Time by Meta-Regression Using Mid-Recruitment Year as a Measure of Time

Supplement 2.
Data Sharing Statement
