# Supplementary material for: Trends in In-Hospital Cardiac Arrest and Mortality Among Children With Cardiac Disease in the Intensive Care Unit: A Systematic Review and Meta-analysis
Source: JAMA Netw Open. 2023 Feb 10;6(2):e2256178. doi: 10.1001/jamanetworkopen.2022.56178 (PMC9918886; doi:10.1001/jamanetworkopen.2022.56178)
Supplement: Supplement 1. — eMethods 1. Detailed Search Strategy eMethods 2. National Heart, Lung, and Blood Institute (NHLBI) Quality Assessment Tool for Observational Studies Checklist eMethods 3. National Heart, Lung, and Blood Institutes (NHLBI) Quality Assessment Tool for Case-Controls Studies Checklist eTable 1. Quality Assessment of Candidate Studies for the Meta-Analysis eTable 2. Pooled Odd Ratios for Risk Factors For In-Hospital Cardiac Arrest (Factors Included in at Least Two Studies) eTable 3. Pooled Odd Ratios for Risk Factors for In-Hospital Mortality After In-Hospital Cardiac Arrest (Factors Included in at Least Two Studies) eFigure 1. Pooled Proportion of Patients Experiencing In-Hospital Cardiac Arrest by Random Effects Meta-Analysis eFigure 2. Pooled Proportion of Patients Experiencing In-Hospital Cardiac Arrest by Random Effects Meta-Analysis According to Last Recruitment Year (<2010, Upper, and ≥2010, Lower) eFigure 3. Pooled Proportion of Patients Experiencing In-Hospital Cardiac Arrest by Random Effects Meta-Analysis in Non-Registry-Based Study (Upper) and in Registry-Based Studies (Lower) eFigure 4. Pooled Proportion of Patients Experiencing In-Hospital Cardiac Arrest by Random Effects Meta-Analysis According to the Category of Patients (Surgical, Upper, and General Cardiac, Lower) eFigure 5. Trend in Incidence of In-Hospital Cardiac Arrest Over Time by Meta-Regression Using Mid-Recruitment Year as a Measure of Time eFigure 6. Pooled Proportion of Patients Who Did Not Achieve Return of Spontaneous Circulation by Random Effects Meta-Analysis eFigure 7. Pooled Proportion of Patients Undergoing Extracorporeal Cardiopulmonary Resuscitation by Random Effects Meta-Analysis eFigure 8. Pooled Proportion of Patients Who Died Suddenly in Centers With Extracorporeal Cardiopulmonary Resuscitation Expertise, by Random Effects Meta-Analysis eFigure 9. In-Hospital Pooled Mortality Rate for In-Hospital Cardiac Arrest by Random Effects Meta-Analysis eFigure 10. In-Hospital Pooled Mortali [file jamanetwopen-e2256178-s001.pdf]

Supplemental Online Content

Sperotto F, Daverio M, Amigoni A, et al. Trends in in-hospital cardiac arrest and mortality among children with cardiac disease in the intensive care unit: a systematic review and meta-analysis. *JAMA Netw Open.* 2023;6(2):e2256178. doi:10.1001/jamanetworkopen.2022.56178

eMethods 1. Detailed Search Strategy

eMethods 2. National Heart, Lung, and Blood Institute (NHLBI) Quality Assessment Tool for Observational Studies Checklist

eMethods 3. National Heart, Lung, and Blood Institutes (NHLBI) Quality Assessment Tool for Case-Controls Studies Checklist

eTable 1. Quality Assessment of Candidate Studies for the Meta-Analysis

eTable 2. Pooled Odd Ratios for Risk Factors For In-Hospital Cardiac Arrest (Factors Included in at Least Two Studies)

eTable 3. Pooled Odd Ratios for Risk Factors for In-Hospital Mortality After In-Hospital Cardiac Arrest (Factors Included in at Least Two Studies)

eFigure 1. Pooled Proportion of Patients Experiencing In-Hospital Cardiac Arrest by Random Effects Meta-Analysis

eFigure 2. Pooled Proportion of Patients Experiencing In-Hospital Cardiac Arrest by Random Effects Meta-Analysis According to Last Recruitment Year (<2010, Upper, and ≥2010, Lower)

eFigure 3. Pooled Proportion of Patients Experiencing In-Hospital Cardiac Arrest by Random Effects Meta-Analysis in Non-Registry-Based Study (Upper) and in Registry-Based Studies (Lower)

eFigure 4. Pooled Proportion of Patients Experiencing In-Hospital Cardiac Arrest by Random Effects Meta-Analysis According to the Category of Patients (Surgical, Upper, and General Cardiac, Lower)

eFigure 5. Trend in Incidence of In-Hospital Cardiac Arrest Over Time by Meta-Regression Using Mid-Recruitment Year as a Measure of Time

eFigure 6. Pooled Proportion of Patients Who Did Not Achieve Return of Spontaneous Circulation by Random Effects Meta-Analysis

eFigure 7. Pooled Proportion of Patients Undergoing Extracorporeal Cardiopulmonary Resuscitation by Random Effects Meta-Analysis

eFigure 8. Pooled Proportion of Patients Who Died Suddenly in Centers With Extracorporeal Cardiopulmonary Resuscitation Expertise, by Random Effects Meta-Analysis

eFigure 9. In-Hospital Pooled Mortality Rate for In-Hospital Cardiac Arrest by Random Effects Meta-Analysis

© 2023 Sperotto F et al. *JAMA Network Open.*

eFigure 10. In-Hospital Pooled Mortality Rate for In-Hospital Cardiac Arrest by Random Effects Meta-Analysis According to Last Recruitment Year (<2010, Upper, and ≥2010, Lower)

eFigure 11. In-Hospital Pooled Mortality Rate by Random Effects Meta-Analysis in Non-Registry-Based Studies (Upper) and in Registry-Based Studies (Lower)

eFigure 12. In-Hospital Pooled Mortality Rate for In-Hospital Cardiac Arrest by Random Effects Meta-Analysis According to Category of Patients (Surgical, Upper, and General Cardiac, Lower)

eFigure 13. Trend in In-Hospital Mortality After In-Hospital Cardiac Arrest Over Time by Meta-Regression Using Mid-Recruitment Year as a Measure of Time

This supplemental material has been provided by the authors to give readers additional information about their work.

© 2023 Sperotto F et al. *JAMA Network Open.*

eMethods 1. Detailed search strategies

Pubmed:

(Resuscitation[mesh:noexp] OR "Cardiopulmonary Resuscitation"[mesh:noexp] OR "Heart Massage"[mesh] OR "Electric Countershock"[mesh] OR resuscitate[tw] OR resuscitation[tw] OR resuscitator[tw] OR resuscitators[tw] OR resuscitative[tw] OR "Cardiopulmonary Resuscitation"[tw] OR CPR[tw] OR "heart massage"[tw] OR "heart massages"[tw] OR "cardio pulmonary resuscitation"[tw] OR "cardio-pulmonary resuscitation"[tw] OR "Code blue"[tw] OR resuscitations[tw] OR resuscitated[tw] OR defibrillate[tw] OR defibrillated[tw] OR defibrillation[tw] OR defibrillations[tw] OR defibrillator[tw] OR defibrillators[tw] OR "chest compression"[tw] OR "chest compressions"[tw] OR "heart compression"[tw] OR "heart compressions"[tw] OR "cardiac compression"[tw] OR "cardiac compressions"[tw]) AND ("Heart Arrest"[mesh:noexp] OR "Heart Arrest"[tw] OR "Cardiac Arrest"[tw] OR "Heart arrests"[tw] OR "cardiac arrests"[tw] OR "Cardiopulmonary Arrest"[tw] OR "cardiorespiratory arrest"[tw] OR "cardiac event"[tw] OR "cardiac events"[tw] OR "poor perfusion"[tw] OR "poor perfusions"[tw] OR "cardiopulmonary arrests"[tw] OR "cardiorespiratory arrests"[tw] OR "cardiopulmonary event"[tw] OR "cardiopulmonary events"[tw] OR "cardiorespiratory event"[tw] OR "cardiorespiratory events"[tw] OR "compromised perfusion"[tw] OR "compromised perfusions"[tw] OR pulseless[tw] OR pulselessness[tw] OR CA[tw] OR CAs[tw] OR CPA[tw] OR CPAs[tw]) AND ("Intensive Care Units"[mesh] OR "Critical Care"[mesh:noexp] OR "Intensive Care Units, Pediatric"[mesh] OR "Intensive Care, Neonatal"[mesh] OR "intensive care"[tw] OR "critical care"[tw] OR "intensive therapy"[tw] OR "intensive treatment"[tw] OR ICU[tw] OR NICU[tw] OR PICU[tw] OR CICU[tw] OR ICUs[tw] OR NICUs[tw] OR PICUs[tw] OR CICUs[tw]) AND ("Heart Diseases"[mesh:noexp] OR "Heart Defects, Congenital"[mesh] OR "Cardiovascular Diseases"[mesh] OR "Vascular Diseases"[mesh:noexp] OR "Heart Defect"[tw] OR "Heart Defects"[tw] OR "Heart Abnormalities"[tw] OR "Heart Abnormality"[tw] OR "congenital heart malformation"[tw] OR "congenital heart malformations"[tw] OR "malformation of the heart"[tw] OR "malformations of the heart"[tw] OR "heart disease"[tw] OR "heart diseases"[tw] OR "cardiac disease"[tw] OR "cardiac diseases"[tw] OR "cardiac abnormality"[tw] OR "cardiac abnormalities"[tw] OR "cardiac malformation"[tw] OR "cardiac malformations"[tw] OR "cardiac pathology"[tw] OR "cardiac pathologies"[tw] OR "heart pathology"[tw] OR "heart pathologies"[tw] OR "heart malformation"[tw] OR "heart malformations"[tw] OR "cardiac conditions"[tw] OR "cardiac condition"[tw] OR "heart condition"[tw] OR "heart conditions"[tw] OR "heart anomaly"[tw] OR "heart anomalies"[tw] OR "cardiac anomaly"[tw] OR "cardiac anomalies"[tw] OR cardiopathy[tw] OR cardopathies[tw] OR "heart deficiency"[tw] OR "heart deficiencies"[tw] OR "heart deformity"[tw] OR "heart deformities"[tw] OR "cardiac deformity"[tw] OR "cardiac deformities"[tw] OR "cardiac disorder"[tw] OR "cardiac disorders"[tw] OR "heart disorder"[tw] OR "heart disorders"[tw] OR "heart dysfunction"[tw] OR "heart dysfunctions"[tw] OR "cardiac dysfunction"[tw] OR "cardiac dysfunctions"[tw] OR angiocardiopathy[tw] OR angiocardiopathies[tw] OR "angiocardiovascular disease"[tw] OR "angiocardiovascular diseases"[tw] OR "Cardiovascular complication"[tw] OR "cardiovascular complications"[tw] OR "heart complication"[tw] OR "heart complications"[tw] OR "cardiovascular disorder"[tw] OR "Cardiovascular disorders"[tw] OR "cardiovascular disturbance"[tw] OR "cardiovascular disturbances"[tw] OR "heart disturbance"[tw] OR "heart disturbances"[tw] OR "Cardiovascular anomaly"[tw] OR "cardiovascular anomalies"[tw] OR "cardiovascular deformity"[tw] OR "cardiovascular deformities"[tw] OR "angiocardiovascular deformity"[tw] OR "angiocardiovascular deformities"[tw] OR "angiocardiovascular anomaly"[tw] OR "angiocardiovascular anomalies"[tw] OR "angiocardiovascular abnormality"[tw] OR "angiocardiovascular abnormalities"[tw] OR "Cardiovascular abnormality"[tw] OR "cardiovascular abnormalities"[tw] OR "cardiovascular dysfunction"[tw] OR "cardiovascular dysfunctions"[tw] OR "angiocardiovascular dysfunction"[tw] OR "angiocardiovascular dysfunctions"[tw] OR "cardiac complication"[tw] OR "cardiac complications"[tw] OR "cardiopulmonary compromise"[tw] OR "cardiorespiratory compromise"[tw] OR "cardiovascular compromise"[tw] OR "cardiac compromise"[tw]) AND (Pediatrics[mesh] OR Child[mesh] OR Infant[mesh] OR Adolescent[mesh] OR Pediatric[tw] OR Pediatrics[tw] OR Paediatric[tw] OR Paediatrics[tw] OR child[tw] OR children[tw] OR infant[tw] OR infants[tw] OR infantile[tw] OR neonate[tw] OR neonates[tw] OR neonatal[tw] OR newborn[tw] OR newborns[tw] OR adolescent[tw] OR adolescents[tw] OR adolescence[tw] OR adolescent[tw] OR youth[tw] OR youths[tw] OR teen[tw] OR teens[tw] OR teenager[tw] OR teenagers[tw] OR baby[tw] OR babies[tw])

Web of Science:

TS=((resuscitate OR resuscitation OR resuscitator OR resuscitators OR resuscitative OR "Cardiopulmonary Resuscitation" OR CPR OR "heart massage" OR "heart massages" OR "cardio pulmonary resuscitation" OR "cardio-pulmonary resuscitation" OR "Code blue" OR resuscitations OR resuscitated OR defibrillate OR defibrillated OR defibrillation OR defibrillations OR defibrillator OR defibrillators OR "chest compression" OR "chest compressions" OR "heart compression" OR "heart compressions" OR "cardiac compression" OR "cardiac compressions") AND ("Heart Arrest" OR "Cardiac Arrest" OR "Heart arrests" OR "cardiac arrests" OR "Cardiopulmonary Arrest" OR "cardiorespiratory arrest" OR "cardiac event" OR "cardiac events" OR "poor perfusion" OR "poor perfusions" OR "cardiopulmonary arrests" OR "cardiorespiratory arrests" OR "cardiopulmonary event" OR "cardiopulmonary events" OR "cardiorespiratory event" OR "cardiorespiratory events" OR "compromised perfusion" OR "compromised perfusions" OR pulseless OR pulselessness OR CA OR CAs OR CPA OR CPAs) AND ("intensive care" OR "critical care" OR "intensive therapy" OR "intensive treatment" OR ICU OR NICU OR PICU OR CICU OR ICUs OR NICUs OR PICUs OR CICUs) AND ("Heart Defect" OR "Heart Defects" OR "Heart Abnormalities" OR

"Heart Abnormality" OR "congenital heart malformation" OR "congenital heart malformations" OR "malformation of the heart" OR "malformations of the heart" OR "heart disease" OR "heart diseases" OR "cardiac disease" OR "cardiac diseases" OR "cardiac abnormality" OR "cardiac abnormalities" OR "cardiac malformation" OR "cardiac malformations" OR "cardiac pathology" OR "cardiac pathologies" OR "heart pathology" OR "heart pathologies" OR "heart malformation" OR "heart malformations" OR "cardiac conditions" OR "cardiac condition" OR "heart condition" OR "heart conditions" OR "heart anomaly" OR "heart anomalies" OR "cardiac anomaly" OR "cardiac anomalies" OR cardiopathy OR cardiopathies OR "heart deficiency" OR "heart deficiencies" OR "heart deformity" OR "heart deformities" OR "cardiac deformity" OR "cardiac deformities" OR "cardiac disorder" OR "cardiac disorders" OR "heart disorder" OR "heart disorders" OR "heart dysfunction" OR "heart dysfunctions" OR "cardiac dysfunction" OR "cardiac dysfunctions" OR angiocardiopathy OR angiocardiopathies OR "angiocardiovascular disease" OR "angiocardiovascular diseases" OR "Cardiovascular complication" OR "cardiovascular complications" OR "heart complication" OR "heart complications" OR "cardiovascular disorder" OR "Cardiovascular disorders" OR "cardiovascular disturbance" OR "cardiovascular disturbances" OR "heart disturbance" OR "heart disturbances" OR "Cardiovascular anomaly" OR "cardiovascular anomalies" OR "cardiovascular deformity" OR "cardiovascular deformities" OR "angiocardiovascular deformity" OR "angiocardiovascular deformities" OR "angiocardiovascular anomaly" OR "angiocardiovascular anomalies" OR "angiocardiovascular abnormality" OR "angiocardiovascular abnormalities" OR "Cardiovascular abnormality" OR "cardiovascular abnormalities" OR "cardiovascular dysfunction" OR "cardiovascular dysfunctions" OR "angiocardiovascular dysfunction" OR "angiocardiovascular dysfunctions" OR "cardiac complication" OR "cardiac complications" OR "cardiopulmonary compromise" OR "cardiorespiratory compromise" OR "cardiovascular compromise" OR "cardiac compromise") AND (Pediatric OR Pediatrics OR Paediatric OR Paediatrics OR child OR children OR infant OR infants OR infantile OR neonate OR neonates OR neonatal OR newborn OR newborns OR adolescent OR adolescents OR adolescence OR adolescent OR youth OR youths OR teen OR teens OR teenager OR teenagers OR baby OR babies))

Embase:

(('resuscitation'/exp OR 'heart massage'/exp OR 'defibrillation'/exp OR resuscitate:ab,ti OR resuscitation:ab,ti OR resuscitator:ab,ti OR resuscitators:ab,ti OR resuscitative:ab,ti OR ‘Cardiopulmonary Resuscitation’:ab,ti OR CPR:ab,ti OR ‘heart massage’:ab,ti OR ‘heart massages’:ab,ti OR ‘cardio pulmonary resuscitation’:ab,ti OR ‘cardio-pulmonary resuscitation’:ab,ti OR ‘Code blue’:ab,ti OR resuscitations:ab,ti OR resuscitated:ab,ti OR defibrillate:ab,ti OR defibrillated:ab,ti OR defibrillation:ab,ti OR defibrillations:ab,ti OR defibrillator:ab,ti OR defibrillators:ab,ti OR ‘chest compression’:ab,ti OR ‘chest compressions’:ab,ti OR ‘heart compression’:ab,ti OR ‘heart compressions’:ab,ti OR ‘cardiac compression’:ab,ti OR ‘cardiac compressions’:ab,ti) AND ('heart arrest'/de OR 'cardiopulmonary arrest'/exp OR ‘Heart Arrest’:ab,ti OR ‘Cardiac Arrest’:ab,ti OR ‘Heart arrests’:ab,ti OR ‘cardiac arrests’:ab,ti OR ‘Cardiopulmonary Arrest’:ab,ti OR ‘cardiorespiratory arrest’:ab,ti OR ‘cardiac event’:ab,ti OR ‘cardiac events’:ab,ti OR ‘poor perfusion’:ab,ti OR ‘poor perfusions’:ab,ti OR ‘cardiopulmonary arrests’:ab,ti OR ‘cardiorespiratory arrests’:ab,ti OR ‘cardiopulmonary event’:ab,ti OR ‘cardiopulmonary events’:ab,ti OR ‘cardiorespiratory event’:ab,ti OR ‘cardiorespiratory events’:ab,ti OR ‘compromised perfusion’:ab,ti OR ‘compromised perfusions’:ab,ti OR pulseless:ab,ti OR pulselessness:ab,ti OR CA:ab,ti OR CAs:ab,ti OR CPA:ab,ti OR CPAs:ab,ti) AND ('intensive care'/de OR 'intensive care unit'/de OR 'neonatal intensive care unit'/exp OR 'pediatric intensive care unit'/exp OR ‘intensive care’:ab,ti OR ‘critical care’:ab,ti OR ‘intensive therapy’:ab,ti OR ‘intensive treatment’:ab,ti OR ICU:ab,ti OR NICU:ab,ti OR PICU:ab,ti OR CICU:ab,ti OR ICUs:ab,ti OR NICUs:ab,ti OR PICUs:ab,ti OR CICUs:ab,ti) AND ('heart disease'/de OR 'congenital heart disease'/de OR 'congenital heart malformation'/de OR 'vascular disease'/de OR 'cardiovascular disease'/de OR ‘Heart Defect’:ab,ti OR ‘Heart Defects’:ab,ti OR ‘Heart Abnormalities’:ab,ti OR ‘Heart Abnormality’:ab,ti OR ‘congenital heart malformation’:ab,ti OR ‘congenital heart malformations’:ab,ti OR ‘malformation of the heart’:ab,ti OR ‘malformations of the heart’:ab,ti OR ‘heart disease’:ab,ti OR ‘heart diseases’:ab,ti OR ‘cardiac disease’:ab,ti OR ‘cardiac diseases’:ab,ti OR ‘cardiac abnormality’:ab,ti OR ‘cardiac abnormalities’:ab,ti OR ‘cardiac malformation’:ab,ti OR ‘cardiac malformations’:ab,ti OR ‘cardiac pathology’:ab,ti OR ‘cardiac pathologies’:ab,ti OR ‘heart pathology’:ab,ti OR ‘heart pathologies’:ab,ti OR ‘heart malformation’:ab,ti OR ‘heart malformations’:ab,ti OR ‘cardiac conditions’:ab,ti OR ‘cardiac condition’:ab,ti OR ‘heart condition’:ab,ti OR ‘heart conditions’:ab,ti OR ‘heart anomaly’:ab,ti OR ‘heart anomalies’:ab,ti OR ‘cardiac anomaly’:ab,ti OR ‘cardiac anomalies’:ab,ti OR cardiopathy:ab,ti OR cardiopathies:ab,ti OR ‘heart deficiency’:ab,ti OR ‘heart deficiencies’:ab,ti OR ‘heart deformity’:ab,ti OR ‘heart deformities’:ab,ti OR ‘cardiac deformity’:ab,ti OR ‘cardiac deformities’:ab,ti OR ‘cardiac disorder’:ab,ti OR ‘cardiac disorders’:ab,ti OR ‘heart disorder’:ab,ti OR ‘heart disorders’:ab,ti OR ‘heart dysfunction’:ab,ti OR ‘heart dysfunctions’:ab,ti OR ‘cardiac dysfunction’:ab,ti OR ‘cardiac dysfunctions’:ab,ti OR angiocardiopathy:ab,ti OR angiocardiopathies:ab,ti OR ‘angiocardiovascular disease’:ab,ti OR ‘angiocardiovascular diseases’:ab,ti OR ‘Cardiovascular complication’:ab,ti OR ‘cardiovascular complications’:ab,ti OR ‘heart complication’:ab,ti OR ‘heart complications’:ab,ti OR ‘cardiovascular disorder’:ab,ti OR ‘Cardiovascular disorders’:ab,ti OR ‘cardiovascular disturbance’:ab,ti OR ‘cardiovascular disturbances’:ab,ti OR ‘heart disturbance’:ab,ti OR ‘heart disturbances’:ab,ti OR ‘Cardiovascular anomaly’:ab,ti OR ‘cardiovascular anomalies’:ab,ti OR ‘cardiovascular deformity’:ab,ti OR ‘cardiovascular deformities’:ab,ti OR ‘angiocardiovascular deformity’:ab,ti OR ‘angiocardiovascular deformities’:ab,ti OR ‘angiocardiovascular anomaly’:ab,ti OR ‘angiocardiovascular anomalies’:ab,ti OR ‘angiocardiovascular abnormality’:ab,ti OR ‘angiocardiovascular abnormalities’:ab,ti OR ‘Cardiovascular abnormality’:ab,ti OR ‘cardiovascular abnormalities’:ab,ti OR ‘cardiovascular dysfunction’:ab,ti OR ‘cardiovascular dysfunctions’:ab,ti OR ‘angiocardiovascular dysfunction’:ab,ti OR ‘angiocardiovascular dysfunctions’:ab,ti OR ‘cardiac complication’:ab,ti OR ‘cardiac complications’:ab,ti OR ‘cardiopulmonary compromise’:ab,ti OR ‘cardiorespiratory compromise’:ab,ti OR ‘cardiovascular compromise’:ab,ti OR ‘cardiac compromise’:ab,ti) AND ('pediatrics'/exp OR 'infant'/exp OR 'adolescent'/exp OR 'child'/exp OR Pediatric:ab,ti OR Pediatrics:ab,ti OR Paediatric:ab,ti OR

© 2023 Sperotto F et al. *JAMA Network Open.*

Paediatrics:ab,ti OR child:ab,ti OR children:ab,ti OR infant:ab,ti OR infants:ab,ti OR infantile:ab,ti OR neonate:ab,ti OR neonates:ab,ti OR neonatal:ab,ti OR newborn:ab,ti OR newborns:ab,ti OR adolescent:ab,ti OR adolescents:ab,ti OR adolescence:ab,ti OR adolescent:ab,ti OR youth:ab,ti OR youths:ab,ti OR teen:ab,ti OR teens:ab,ti OR teenager:ab,ti OR teenagers:ab,ti OR baby:ab,ti OR babies:ab,ti))

CINAHL:

((MH ‘Resuscitation’) OR (MH ‘Resuscitation, Cardiopulmonary’) OR (MH ‘Heart Massage’) OR (MH ‘Defibrillation’) OR TX resuscitate OR TX resuscitation OR TX resuscitator OR TX resuscitators OR TX resuscitative OR TX ‘Cardiopulmonary Resuscitation’ OR TX CPR OR TX ‘heart massage’ OR TX ‘heart massages’ OR TX ‘cardio pulmonary resuscitation’ OR TX ‘cardio-pulmonary resuscitation’ OR TX ‘Code blue’ OR TX resuscitations OR TX resuscitated OR TX defibrillate OR TX defibrillated OR TX defibrillation OR TX defibrillations OR TX defibrillator OR TX defibrillators OR TX ‘chest compression’ OR TX ‘chest compressions’ OR TX ‘heart compression’ OR TX ‘heart compressions’ OR TX ‘cardiac compression’ OR TX ‘cardiac compressions’) AND ((MH ‘Heart Arrest’) OR TX ‘Heart Arrest’ OR TX ‘Cardiac Arrest’ OR TX ‘Heart arrests’ OR TX ‘cardiac arrests’ OR TX ‘Cardiopulmonary Arrest’ OR TX ‘cardiorespiratory arrest’ OR TX ‘cardiac event’ OR TX ‘cardiac events’ OR TX ‘poor perfusion’ OR TX ‘poor perfusions’ OR TX ‘cardiopulmonary arrests’ OR TX ‘cardiorespiratory arrests’ OR TX ‘cardiopulmonary event’ OR TX ‘cardiopulmonary events’ OR TX ‘cardiorespiratory event’ OR TX ‘cardiorespiratory events’ OR TX ‘compromised perfusion’ OR TX ‘compromised perfusions’ OR TX pulseless OR TX pulselessness OR TX ‘CA’ OR TX CAs OR TX CPA OR TX CPAs) AND ((MH ‘Critical Care’) OR (MH ‘Intensive Care, Neonatal’) OR (MH ‘Intensive Care Units+’) OR TX ‘intensive care’ OR TX ‘critical care’ OR TX ‘intensive therapy’ OR TX ‘intensive treatment’ OR TX ICU OR TX NICU OR TX PICU OR TX CICU OR TX ICUs OR TX NICUs OR TX PICUs OR TX CICUs) AND ((MH ‘Heart Diseases) OR (MH ‘Vascular Diseases’) OR TX ‘Heart Defect’ OR TX ‘Heart Defects’ OR TX ‘Heart Abnormalities’ OR TX ‘Heart Abnormality’ OR TX ‘congenital heart malformation’ OR TX ‘congenital heart malformations’ OR TX ‘malformation of the heart’ OR TX ‘malformations of the heart’ OR TX ‘heart disease’ OR TX ‘heart diseases’ OR TX ‘cardiac disease’ OR TX ‘cardiac diseases’ OR TX ‘cardiac abnormality’ OR TX ‘cardiac abnormalities’ OR TX ‘cardiac malformation’ OR TX ‘cardiac malformations’ OR TX ‘cardiac pathology’ OR TX ‘cardiac pathologies’ OR TX ‘heart pathology’ OR TX ‘heart pathologies’ OR TX ‘heart malformation’ OR TX ‘heart malformations’ OR TX ‘cardiac conditions’ OR TX ‘cardiac condition’ OR TX ‘heart condition’ OR TX ‘heart conditions’ OR TX ‘heart anomaly’ OR TX ‘heart anomalies’ OR TX ‘cardiac anomaly’ OR TX ‘cardiac anomalies’ OR TX cardiopathy OR TX cardiopathies OR TX ‘heart deficiency’ OR TX ‘heart deficiencies’ OR TX ‘heart deformity’ OR TX ‘heart deformities’ OR TX ‘cardiac deformity’ OR TX ‘cardiac deformities’ OR TX ‘cardiac disorder’ OR TX ‘cardiac disorders’ OR TX ‘heart disorder’ OR TX ‘heart disorders’ OR TX ‘heart dysfunction’ OR TX ‘heart dysfunctions’ OR TX ‘cardiac dysfunction’ OR TX ‘cardiac dysfunctions’ OR TX angiocardiopathy OR TX angiocardiopathies OR TX ‘angiocardiovascular disease’ OR TX ‘angiocardiovascular diseases’ OR TX ‘Cardiovascular complication’ OR TX ‘cardiovascular complications’ OR TX ‘heart complication’ OR TX ‘heart complications’ OR TX ‘cardiovascular disorder’ OR TX ‘Cardiovascular disorders’ OR TX ‘cardiovascular disturbance’ OR TX ‘cardiovascular disturbances’ OR TX ‘heart disturbance’ OR TX ‘heart disturbances’ OR TX ‘Cardiovascular anomaly’ OR TX ‘cardiovascular anomalies’ OR TX ‘cardiovascular deformity’ OR TX ‘cardiovascular deformities’ OR TX ‘angiocardiovascular deformity’ OR TX ‘angiocardiovascular deformities’ OR TX ‘angiocardiovascular anomaly’ OR TX ‘angiocardiovascular anomalies’ OR TX ‘angiocardiovascular abnormality’ OR TX ‘angiocardiovascular abnormalities’ OR TX ‘Cardiovascular abnormality’ OR TX ‘cardiovascular abnormalities’ OR TX ‘cardiovascular dysfunction’ OR TX ‘cardiovascular dysfunctions’ OR TX ‘angiocardiovascular dysfunction’ OR TX ‘angiocardiovascular dysfunctions’ OR TX ‘cardiac complication’ OR TX ‘cardiac complications’ OR TX ‘cardiopulmonary compromise’ OR TX ‘cardiorespiratory compromise’ OR TX ‘cardiovascular compromise’ OR TX ‘cardiac compromise’) AND ((MH ‘Pediatrics+’) OR (MH ‘Child’) OR (MH ‘Infant’) OR (MH ‘Infant, Newborn+’) OR (MH ‘Child, Hospitalized’) OR (MH ‘Child, Preschool’) OR (MH ‘Child, Medically Fragile’) OR (MH ‘Adolescence+’) OR TX Pediatric OR TX Pediatrics OR TX Paediatric OR TX Paediatrics OR TX child OR TX children OR TX infant OR TX infants OR TX infantile OR TX neonate OR TX neonates OR TX neonatal OR TX newborn OR TX newborns OR TX adolescent OR TX adolescents OR TX adolescence OR TX adolescent OR TX youth OR TX youths OR TX teen OR TX teens OR TX teenager OR TX teenagers OR TX baby OR TX babies)

© 2023 Sperotto F et al. *JAMA Network Open.*

eMethods 2. National Heart, Lung, and Blood Institute (NHLBI) Quality Assessment Tool for Observational Cohort Studies checklist

Criteria	Yes	No	Other (CD, NR, NA)*
1. Was the research question or objective in this paper clearly stated?			
2. Was the study population clearly specified and defined?			
3. Was the participation rate of eligible persons at least 50%?			
4. Were all the subjects selected or recruited from the same or similar populations (including the same time period)? Were inclusion and exclusion criteria for being in the study prespecified and applied uniformly to all participants?			
5. Was a sample size justification, power description, or variance and effect estimates provided?			
6. For the analyses in this paper, were the exposure(s) of interest measured prior to the outcome(s) being measured?			
7. Was the timeframe sufficient so that one could reasonably expect to see an association between exposure and outcome if it existed?			
8. For exposures that can vary in amount or level, did the study examine different levels of the exposure as related to the outcome (e.g., categories of exposure, or exposure measured as continuous variable)?			
9. Were the exposure measures (independent variables) clearly defined, valid, reliable, and implemented consistently across all study participants?			
10. Was the exposure(s) assessed more than once over time?			
11. Were the outcome measures (dependent variables) clearly defined, valid, reliable, and implemented consistently across all study participants?			
12. Were the outcome assessors blinded to the exposure status of participants?			
13. Was loss to follow-up after baseline 20% or less?			
14. Were key potential confounding variables measured and adjusted statistically for their impact on the relationship between exposure(s) and outcome(s)?			

*CD, cannot determine; NA, not applicable; NR, not reported
https://www.nhlbi.nih.gov/health-topics/study-quality-assessment-tools

© 2023 Sperotto F et al. *JAMA Network Open.*

eMethods 3. National Heart, Lung, and Blood Institute (NHLBI) Quality Assessment Tool for Case-Control Studies checklist

Criteria	Yes	No	Other (CD, NR, NA)*
1. Was the research question or objective in this paper clearly stated and appropriate?			
2. Was the study population clearly specified and defined?			
3. Did the authors include a sample size justification?			
4. Were controls selected or recruited from the same or similar population that gave rise to the cases (including the same timeframe)?			
5. Were the definitions, inclusion and exclusion criteria, algorithms or processes used to identify or select cases and controls valid, reliable, and implemented consistently across all study participants?			
6. Were the cases clearly defined and differentiated from controls?			
7. If less than 100 percent of eligible cases and/or controls were selected for the study, were the cases and/or controls randomly selected from those eligible?			
8. Was there use of concurrent controls?			
9. Were the investigators able to confirm that the exposure/risk occurred prior to the development of the condition or event that defined a participant as a case?			
10. Were the measures of exposure/risk clearly defined, valid, reliable, and implemented consistently (including the same time period) across all study participants?			
11. Were the assessors of exposure/risk blinded to the case or control status of participants?			
12. Were key potential confounding variables measured and adjusted statistically in the analyses? If matching was used, did the investigators account for matching during study analysis?			

*CD, cannot determine; NA, not applicable; NR, not reported
https://www.nhlbi.nih.gov/health-topics/study-quality-assessment-tools

© 2023 Sperotto F et al. *JAMA Network Open.*

eTable 1. Quality assessment of candidate studies for the meta-analysis

Author	Year	Q1	Q2	Q3	Q4	Q5	Q6	Q7	Q8	Q9	Q10	Q11	Q12	Q13	Q14	Reviewer 1 (M.D.)	Reviewer 2 (A.A.)	Agreement
**Cohort studies (14-item NHLBI Quality Assessment Tool for Observational Cohort Studies)**																		
**Yates A**	**2019**	Y	Y	Y	Y	N/A	Y	Y	N/A	Y	N/A	Y	N/A	Y	N	Good	Good	Good
**Dagan M**	**2019**	Y	Y	Y	Y	N/A	Y	Y	Y	Y	Y	Y	N/A	Y	Y	Good	Good	Good
**Dhillon GS**	**2018**	Y	Y	Y	Y	N/A	Y	Y	Y	Y	Y	Y	N/A	Y	Y	Good	Good	Good
**Alten JA**	**2017**	Y	Y	Y	Y	N/A	Y	Y	N/A	Y	Y	Y	N/A	N	Y	Good	Good	Good
**Berg RA**	**2016**	Y	Y	Y	Y	N/A	Y	Y	Y	Y	Y	Y	N/A	Y	Y	Good	Good	Good
**Gupta P**	**2016**	Y	Y	Y	Y	N/A	Y	Y	N/A	Y	Y	Y	N/A	Y	Y	Good	Good	Good
**Butts RJ**	**2014**	Y	Y	Y	Y	N	Y	Y	Y	Y	Y	Y	N/A	Y	Y	Good	Good	Good
**Gupta P** *(Ann T S)*	**2014**	Y	Y	Y	Y	N/A	Y	Y	Y	Y	N/A	Y	N/A	Y	Y	Good	Good	Good
**Gupta P** *(PCCM)*	**2014**	Y	Y	Y	Y	N/A	Y	Y	Y	Y	Y	Y	N/A	Y	Y	Good	Good	Good
**Ahmadi**	**2013**	Y	Y	Y	Y	N/A	Y	Y	Y	N	N/A	Y	N/A	Y	N	Poor	Poor	Poor
**Argawal HA**	**2012**	Y	Y	Y	Y	N/A	Y	Y	N/A	N/A	N/A	Y	N/A	N/A	N	Fair	Fair	Fair
**Gaies MG**	**2012**	Y	Y	Y	Y	N/A	Y	Y	Y	Y	Y	Y	N/A	Y	Y	Good	Good	Good
**Dorfman AT**	**2008**	Y	Y	Y	Y	N/A	Y	Y	Y	Y	Y	Y	N/A	Y	N	Fair	Fair	Fair
**Gillespie M**	**2006**	Y	Y	Y	Y	N/A	Y	Y	Y	Y	N/A	Y	N/A	N/A	Y	Fair	Good	Fair
**Brown KL**	**2003**	Y	Y	Y	Y	N/A	Y	Y	Y	Y	N/A	Y	N/A	N/A	Y	Fair	Good	Fair
**Parra DA**	**2000**	Y	Y	Y	Y	N/A	Y	Y	Y	Y	N/A	Y	N/A	Y	N	Good	Fair	Fair
**Rhodes J**	**1999**	Y	Y	Y	Y	N/A	Y	Y	Y	Y	N/A	Y	N/A	Y	N	Fair	Fair	Fair
**Case Control Studies (12-item NHLBI Quality Assessment Tool for Case Control Studies)**																		
**Hansen G**	**2011**	Y	Y	N/A	Y	Y	Y	N/A	Y	Y	N/A	Y	N/A	-	-	Good	Good	Good
**Suominen P**	**2001**	Y	Y	N/A	Y	Y	Y	N/A	Y	Y	N/A	Y	N/A	-	-	Fair	Fair	Fair

Q1-Q14 refer to the question included in the NHLBI quality assessment tool for observational cohort studies and the NHLBI quality assessment tool for case control studies, reported in the Supplemental Methods 2. Raters followed the detailed instructions provided by this tool (https://www.nhlbi.nih.gov/health-topics/study-quality-assessment-tools).

© 2023 Sperotto F et al. *JAMA Network Open.*

eTable 2. Pooled Odd Ratios for risk factors for in-hospital cardiac arrest (factors included in at least 2 studies)

Variable and studies included	Crude OR (95% CI)	Exposure group N	Control group N	Ht I^2	Pooled-OR (95% CI) by random effects meta-analysis	
					DerSimonian-Laird estimator	Paule-Mandel estimator
**Neonatal age**		23363	62815	84%	**3.44 (2.64-4.48)**	**3.44 (2.64-4.48)**
Alten JA, 2017	2.97 (2.46-3.58)					
Gupta P, 2014 ATS	3.89 (3.53-4.27)					
**Prematurity**		7348	83787	95%	**2.21 (1.16-4.21)**	**2.21 (1.19-4.11)**
Dagan M, 2019	1.89 (1.35-2.66)					
Alten JA, 2017	4.11 (2.99-5.65)					
Gupta P, 2014 ATS	1.43 (1.24-1.64)					
**Gender, male**		64864	53694	62%	0.91 (0.79-1.06)	0.90 (0.73-1.12)
Dagan M, 2019	0.62 (0.47-0.81)					
Alten JA, 2017	1.03 (0.86-1.23)					
Gupta P, 2016	0.94 (0.81-1.09)					
Gupta P, 2014 ATS	0.96 (0.88-1.06)					
Hansen G, 2011	0.56 (0.23-1.40)					
Suominen P, 2001	1.45 (0.78-2.68)					
**Genetic disorder/syndrome**		28655	89476	65%	**1.42 (1.20-1.69)**	**1.42 (1.19-1.70)**
Dagan M, 2019	1.97 (1.41-2.73)					
Alten JA, 2017	1.29 (1.05-1.58)					
Gupta P, 2016	1.18 (0.98-1.41)					
Gupta P, 2014 ATS	1.54 (1.40-1.70)					
Hansen G, 2011	1.00 (0.17-5.81)					
**Single ventricle physiology**		4503	23521	0%	**2.44 (2.09-2.85)**	**2.44 (2.09-2.85)**
Gupta P, 2016	2.46 (2.09-2.88)					
Suominen P, 2001	2.26 (1.26-4.06)					
**Heart failure**		7049	26358	99%	9.30 (0.99-87.26)	**9.30 (1.02-84.95)**
Alten JA, 2017	3.00 (2.21-4.08)					
Gupta P, 2016	28.67 (22.93-35.86)					
**Arrhythmia**		6878	35939	94%	**2.95 (1.77-4.91)**	**2.95 (1.77-4.91)**
Alten JA, 2017	2.26 (1.84-2.78)					
Gupta P, 2016	3.80 (3.28-4.42)					
**Pulmonary hypertension**		2049	40768	0%	**3.28 (2.76-3.89)**	**3.28 (2.76-3.89)**
Alten JA, 2017	3.01 (2.24-4.05)					
Gupta P, 2016	3.43 (2.78-4.22)					
**Renal failure**		1498	95681	98%	**4.64 (1.36-15.86)**	**4.64 (1.39-15.55)**
Gupta P, 2016	8.55 (6.99-10.46)					
Gupta P, 2014 ATS	2.49 (1.82-3.40)					
**Sepsis**		1410	95769	92%	**2.91 (1.27-6.66)**	**2.91 (1.29-6.60)**
Gupta P, 2016	4.46 (3.11-6.38)					
Gupta P, 2014 ATS	1.93 (1.46-2.56)					
**Seizures**		805	26191	61%	**5.22 (2.22-12.28)**	**5.25 (2.17-12.14)**
Gupta P, 2016	7.00 (5.65-8.66)					
Hansen G, 2011	2.78 (0.89-8.63)					
**Mechanical Ventilation**		14679	89448	97%	**4.78 (2.85-8.02)**	**4.76 (3.13-7.21)**
Alten JA, 2017	5.38 (4.00-7.27)					
Gupta P, 2016	6.43 (5.51-7.51)					
Gupta P, 2014 ATS	3.20 (2.90-3.53)					
**Pre-arrest ECMO**		1576	46285	97%	**15.92 (5.69-44.54)**	**15.85 (5.88-42.78)**
Dagan M, 2019	25.61 (18.50-35.43)					
Alten JA, 2017	4.02 (2.85-5.68)					
Gupta P, 2016	26.91 (22.91-31.74)					
Hansen G, 2011	34.83 (4.20-288.64)					
**Deep Hypothermic Circulatory Arrest**		153	325	0%	**2.16 (1.20-3.91)**	**2.16 (1.20-3.91)**
Hansen G, 2011	2.26 (0.58-8.75)					
Suominen P, 2001	2.14 (1.11-4.13)					
**High STS mortality category 4-5**		25369	81054	95%	**5.31 (3.55-7.92)**	**5.30 (3.70-7.61)**
Alten JA, 2017	6.13 (4.81-7.80)					
Gupta P, 2016	3.70 (3.19-4.29)					
Gupta P, 2014 ATS	6.61 (6.00-7.29)					

Pooled Odd Ratios were computed by random effects meta-analysis using the Mantel-Haenszel method with the DerSimonian-Laird and the Paule-Mandel variance estimators, and are reported as OR (95% confidence interval).
ECMO: extracorporeal membrane oxygenation; Ht: heterogeneity; OR: odds ratio; STS: Society of Thoracic Surgeons.

© 2023 Sperotto F et al. *JAMA Network Open.*

eTable 3. Pooled Odd Ratios for risk factors for in-hospital mortality after in-hospital cardiac arrest (factors included in at least two studies)

Variable and studies included	Crude OR (95% CI)	Exposure group N	Control group N	Ht I^2	Pooled-OR (95% CI) by random effects meta-analysis	
					DerSimonian-Laird estimator	Paule-Mandel estimator
**Neonatal age**						
Alten JA, 2017	0.83 (0.58-1.20)	225	369	0%	0.84 (0.60-1.19)	0.84 (0.60-1.19)
Gaies MG, 2012	0.32 (0.32-2.90)					
**High STS mortality category (4-5)**						
Alten JA, 2017	1.90 (1.14-3.16)	603	438	0%	**2.19 (1.66-2.89)**	**2.19 (1.66-2.88)**
Gupta P, 2016	2.33 (1.68-3.24)					

Pooled Odd Ratios were computed by random effects meta-analysis using the Mantel-Haenszel method with the DerSimonian-Laird and the Paule-Mandel variance estimators, and are reported as OR (95% confidence interval).
ECMO: extracorporeal membrane oxygenation; Ht: heterogeneity; OR: odds ratio; STS: Society of Thoracic Surgeons.

© 2023 Sperotto F et al. *JAMA Network Open.*

eFigure 1. Pooled proportion of patients experiencing in-hospital cardiac arrest by random effects meta-analysis

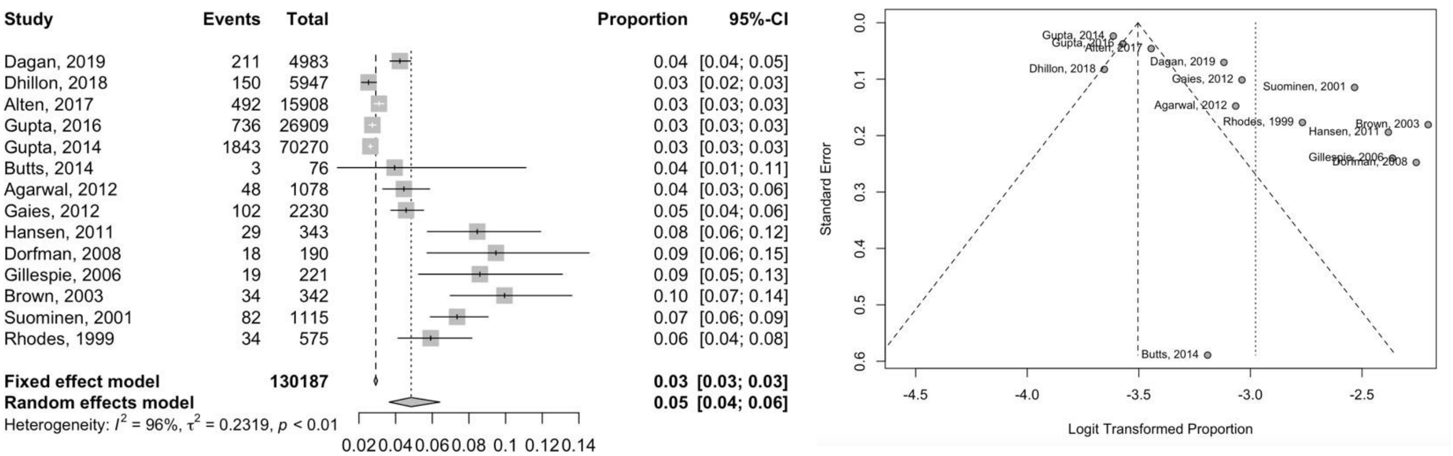

eFigure 2. Pooled proportion of patients experiencing in-hospital cardiac arrest by random effects meta-analysis according to last recruitment year (<2010, upper, and ≥2010, lower)

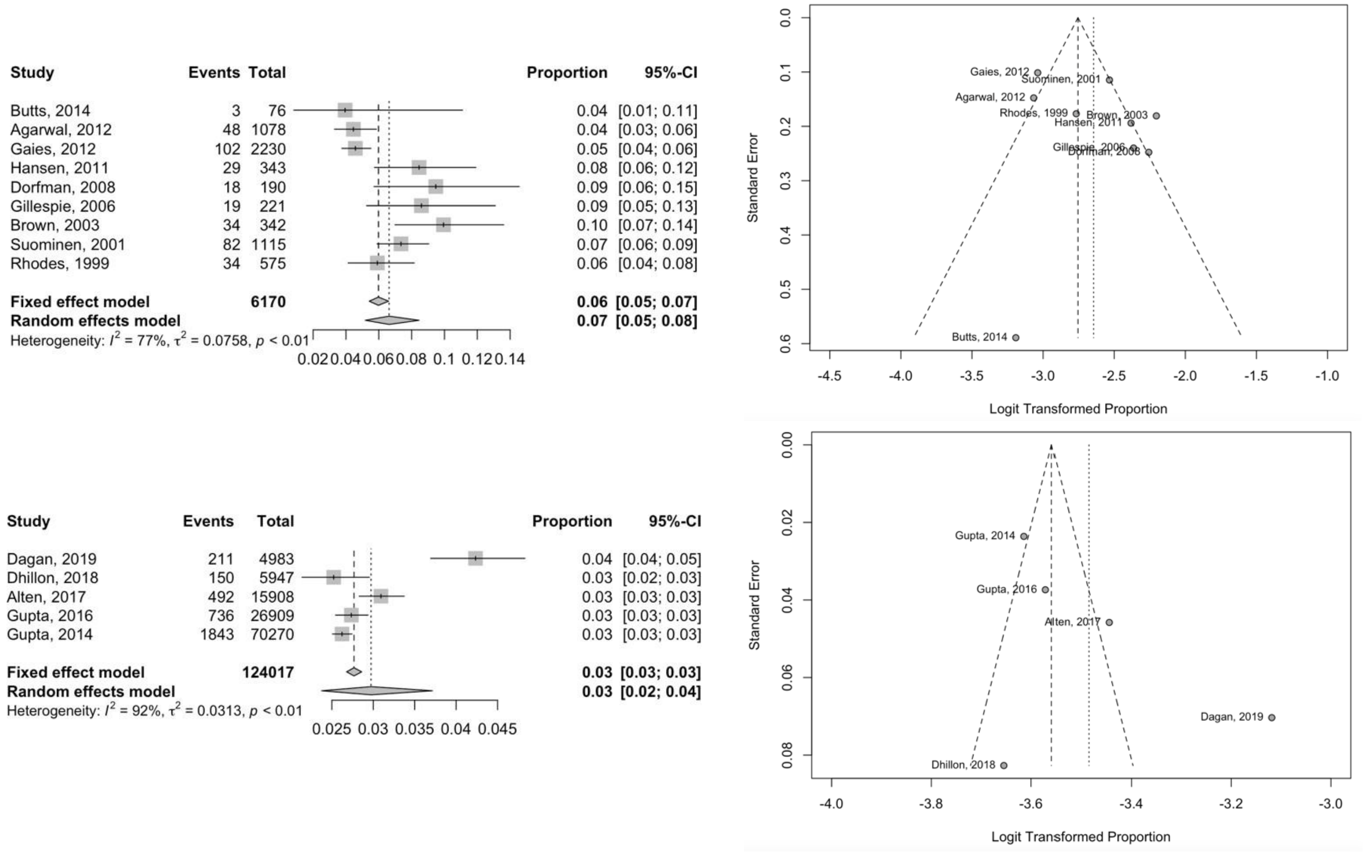

© 2023 Sperotto F et al. *JAMA Network Open.*

eFigure 3. Pooled proportion of patients experiencing in-hospital cardiac arrest by random effects meta-analysis in non-registry-based study (upper) and in registry-based studies (lower)

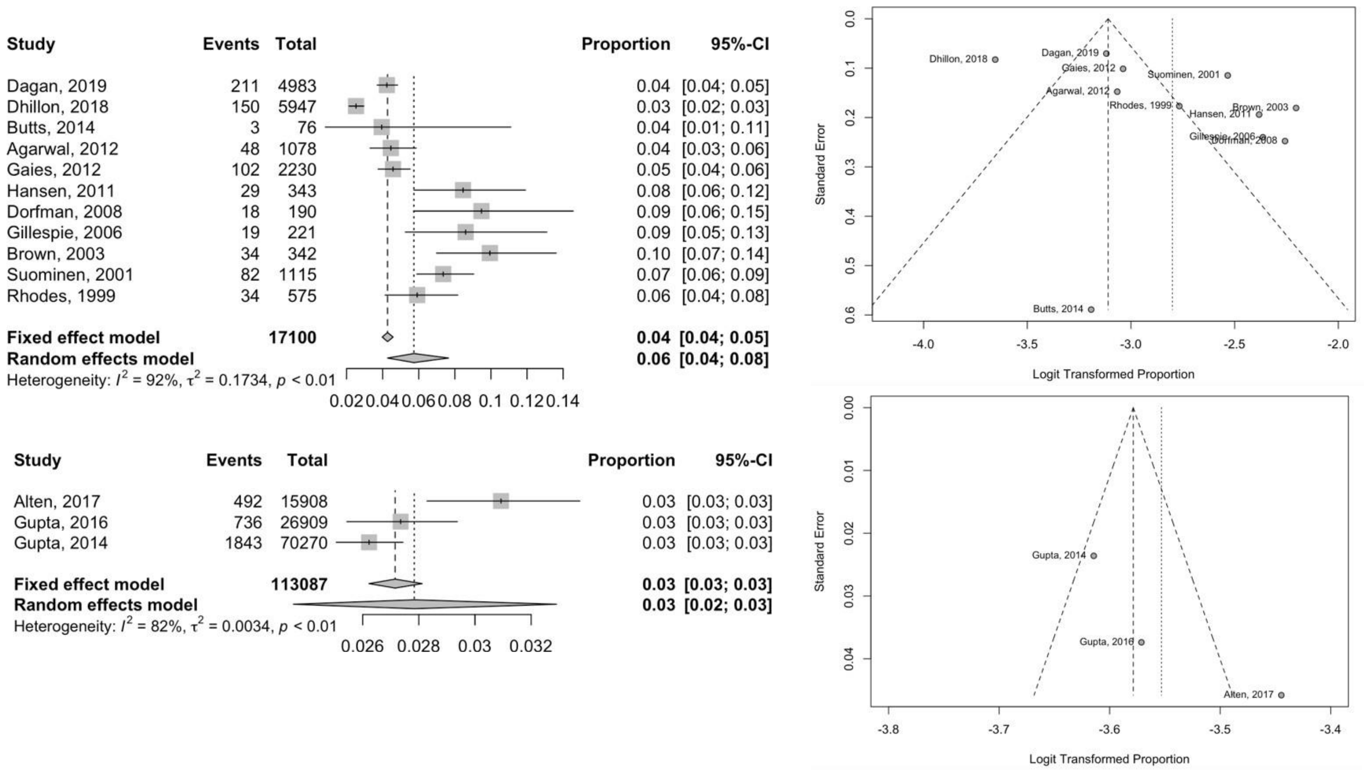

eFigure 4. Pooled proportion of patients experiencing in-hospital cardiac arrest by random effects meta-analysis according to the category of patients (surgical, upper, and general cardiac, lower)

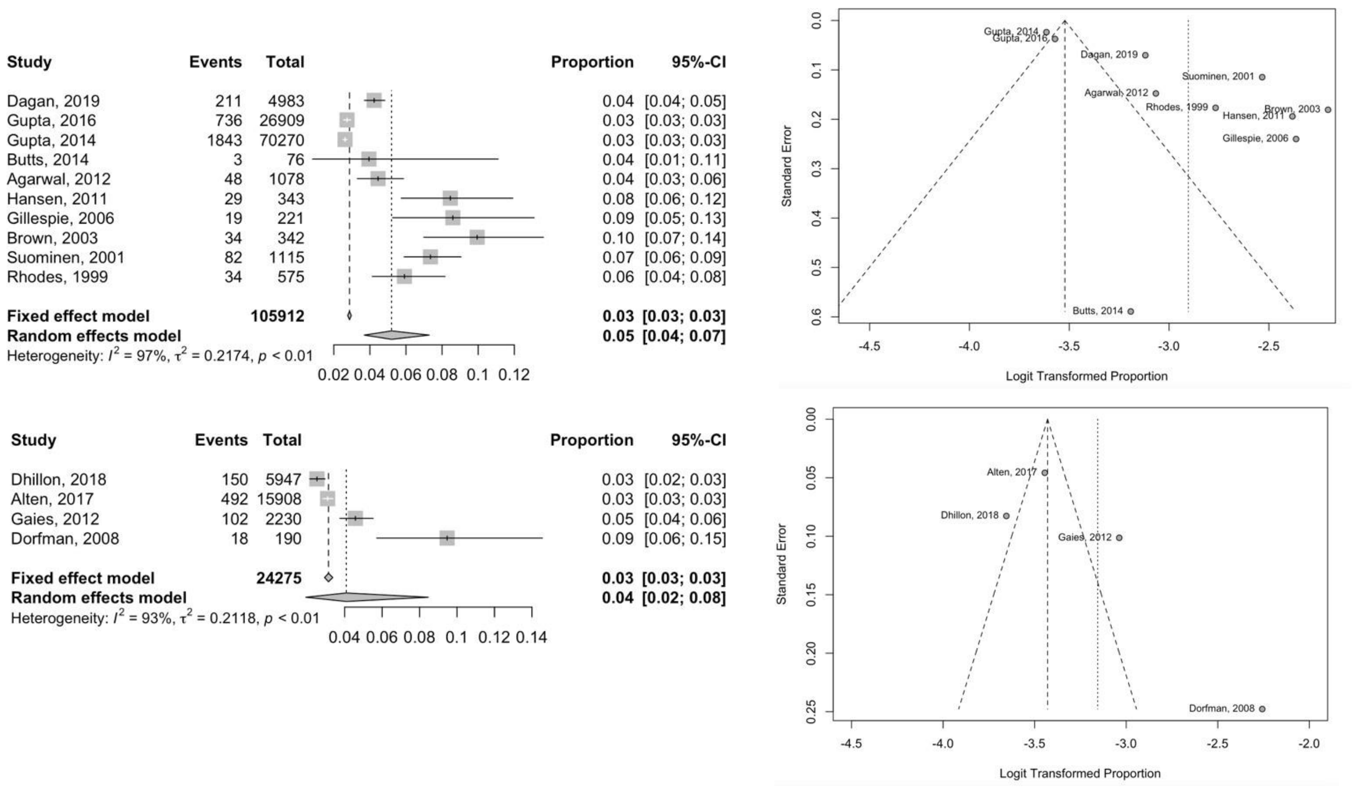

© 2023 Sperotto F et al. *JAMA Network Open.*

eFigure 5. Trend in incidence of in-hospital cardiac arrest over time by meta-regression using mid-recruitment year as a measure of time. The incidence of in-hospital cardiac arrest in critically ill pediatric patients with cardiac disease significantly decreased in the last 20 years (p<0.001). The model was adjusted for type of study (registry-based vs non-registry-based) and diagnostic category (surgical vs general cardiac).

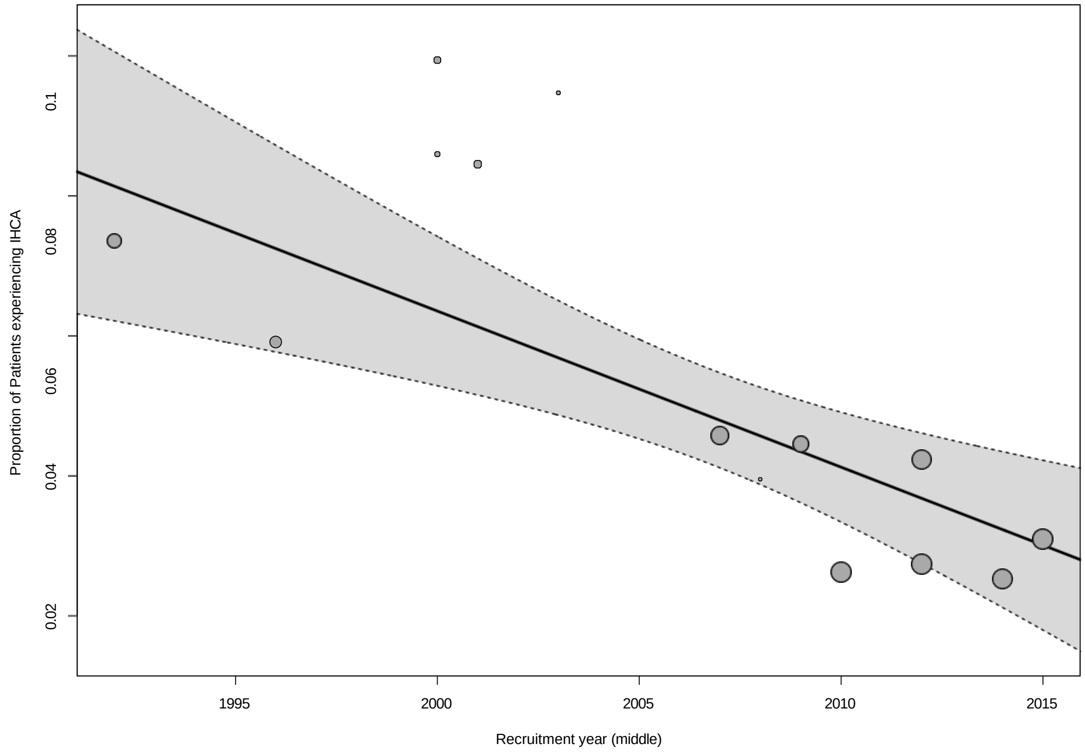

eFigure 7. Pooled proportion of patients who did not achieve return of spontaneous circulation by random effects meta-analysis

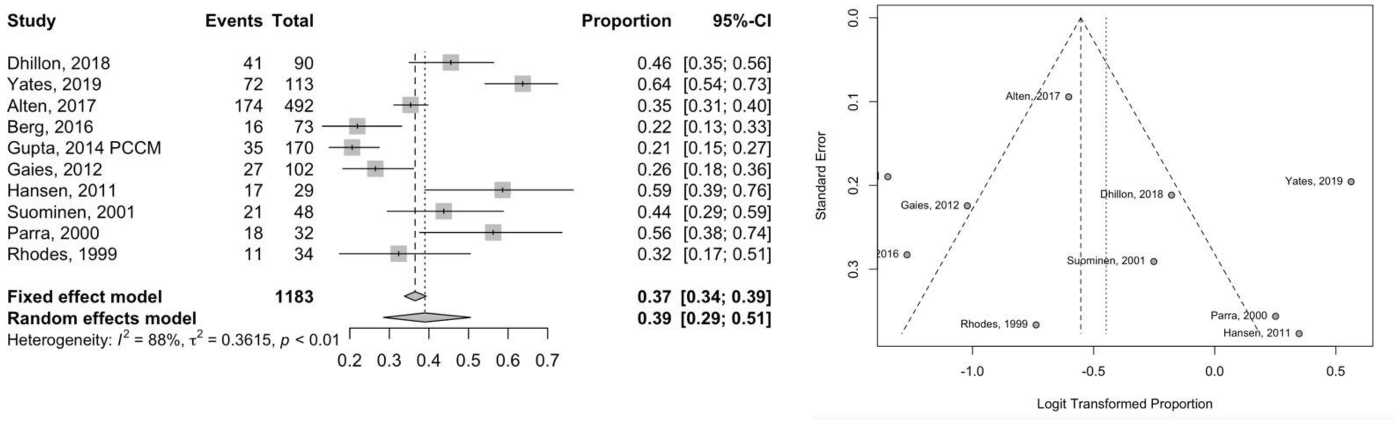

© 2023 Sperotto F et al. *JAMA Network Open.*

eFigure 6. Pooled proportion of patients undergoing extracorporeal cardiopulmonary resuscitation by random effects meta- analysis

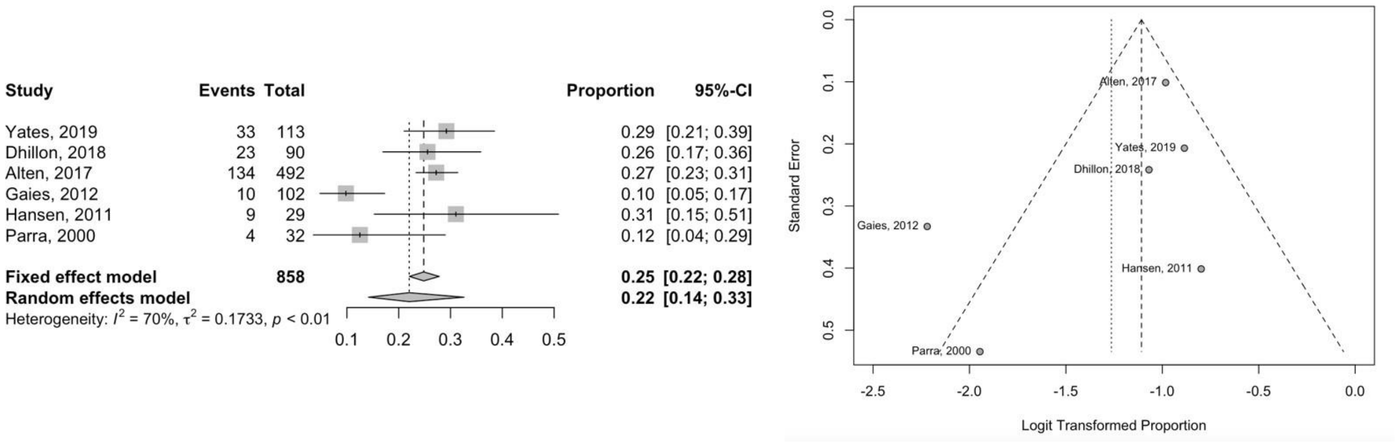

eFigure 8. Pooled proportion of patients who died suddenly in centers with extracorporeal cardiopulmonary resuscitation expertise, by random effects meta-analysis

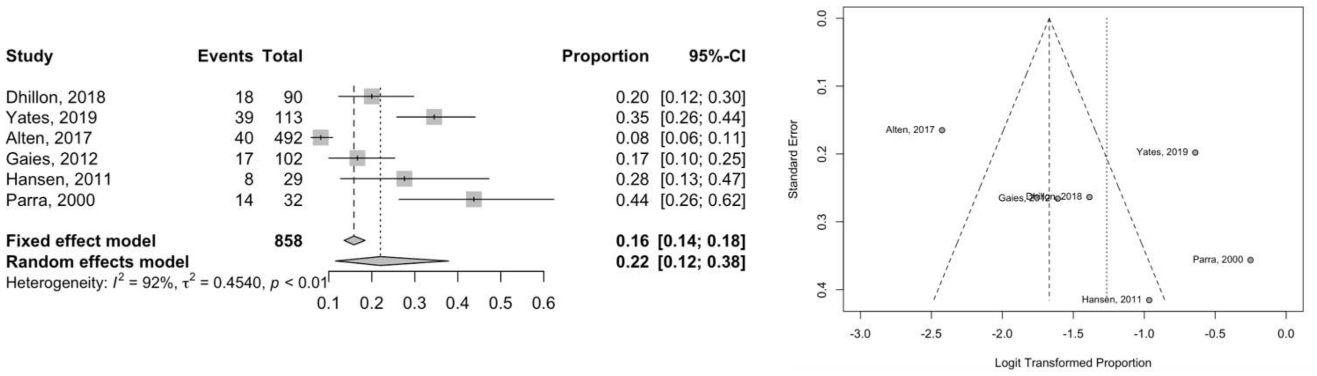

eFigure 9. In-hospital pooled mortality rate for in-hospital cardiac arrest by random effects meta-analysis

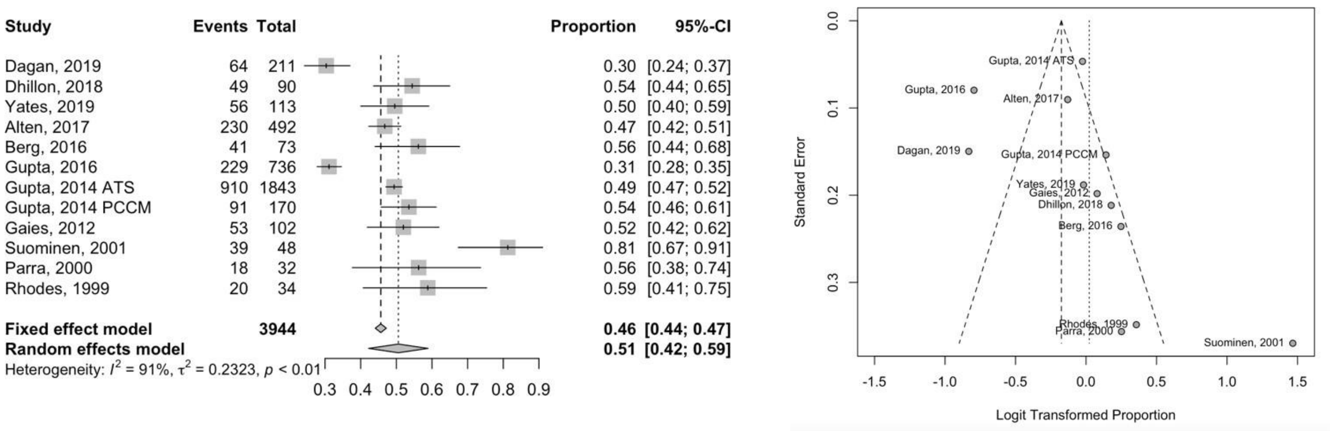

© 2023 Sperotto F et al. *JAMA Network Open.*

eFigure 10. In-hospital pooled mortality rate for in-hospital cardiac arrest by random effects meta-analysis according to last recruitment year (<2010, upper, and ≥2010, lower)

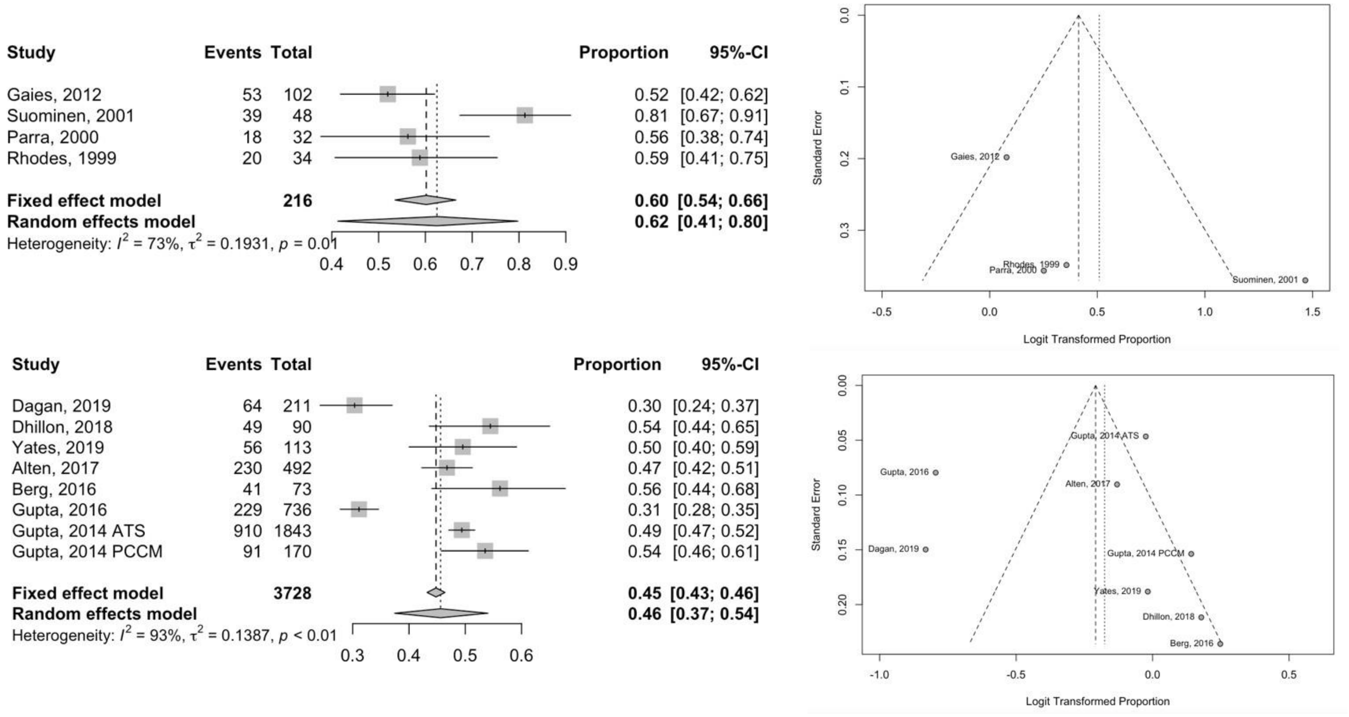

eFigure 11. In-hospital pooled mortality rate by random effects meta-analysis in non- registry-based studies (upper) and in registry-based studies (lower)

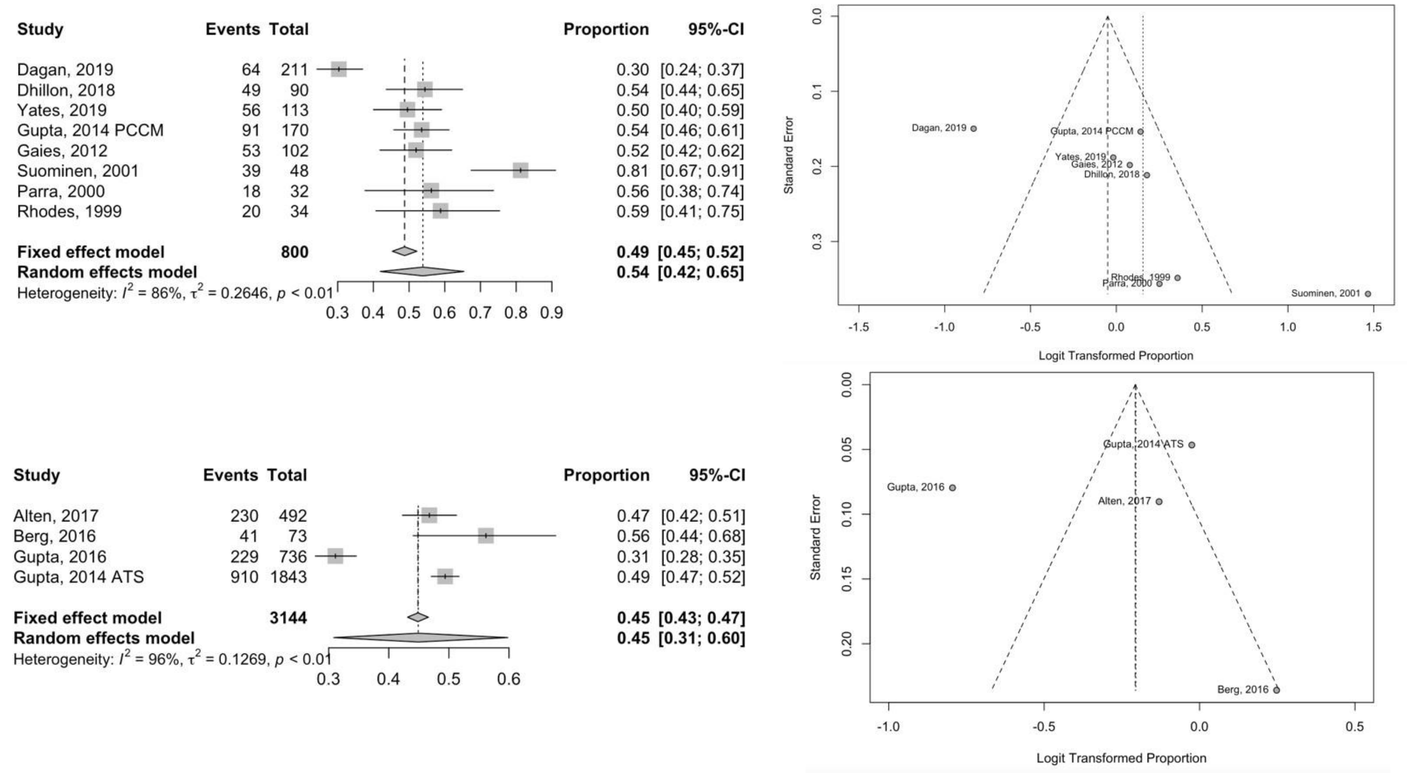

© 2023 Sperotto F et al. *JAMA Network Open.*

eFigure 12. In-hospital pooled mortality rate for in-hospital cardiac arrest by random effects meta-analysis according to category of patients (surgical, upper, and general cardiac, lower)

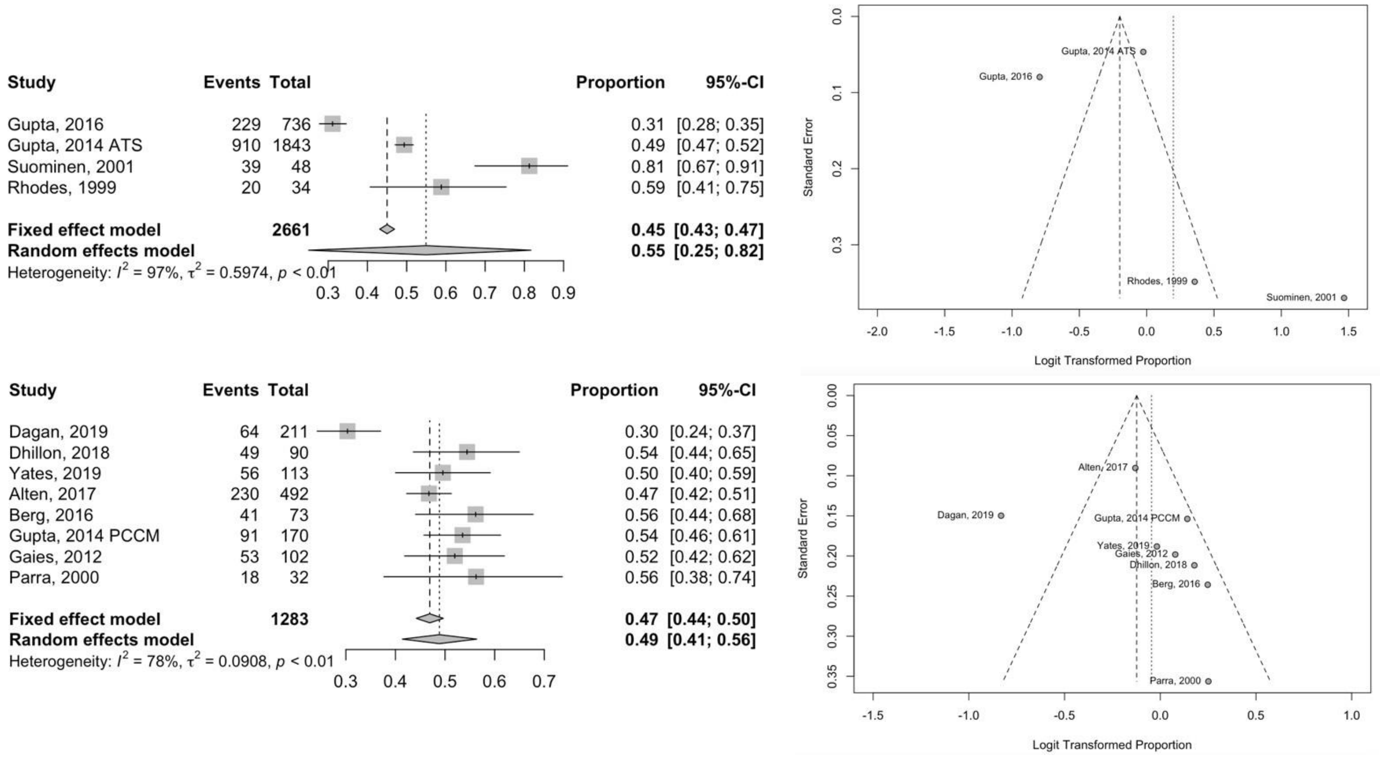

eFigure 13. Trend in in-hospital mortality after in-hospital cardiac arrest over time by meta-regression using mid-recruitment year as a measure of time. The in-hospital mortality rate after in-hospital cardiac arrest in critically ill pediatric patients with cardiac disease significantly decreased in the last 20 years (p<0.001). The model was adjusted for diagnostic category (surgical vs general cardiac).

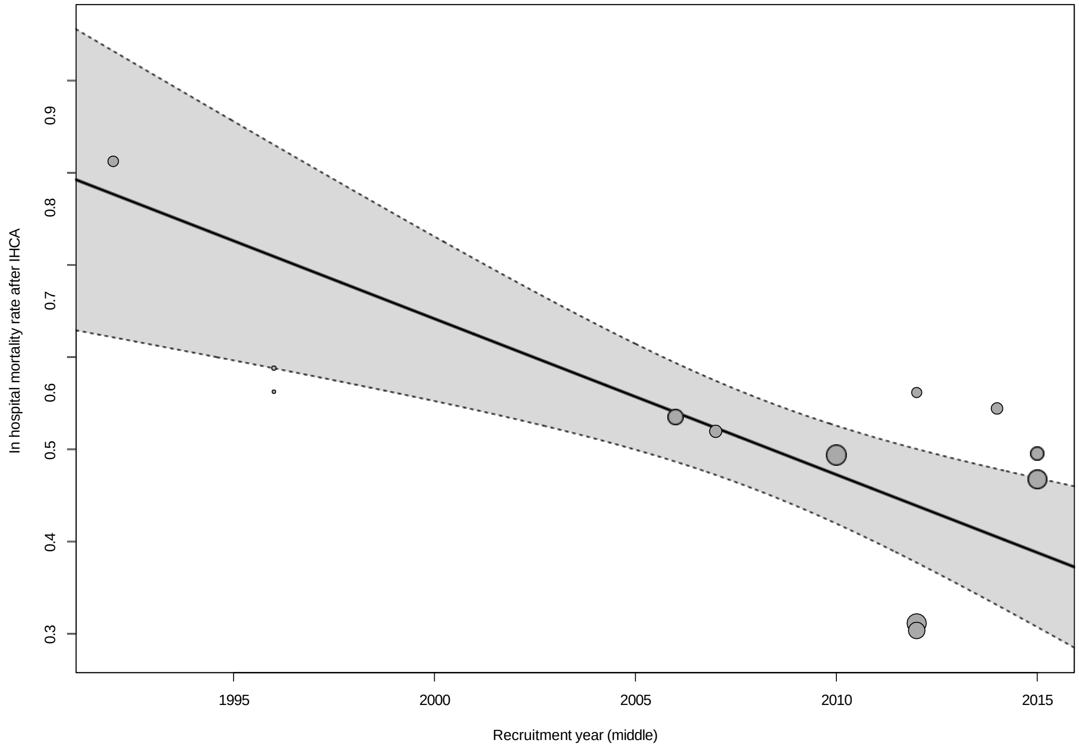

© 2023 Sperotto F et al. *JAMA Network Open.*
