# Supplementary material for: Trends in In-Hospital Cardiac Arrest and Mortality Among Children With Cardiac Disease in the Intensive Care Unit: A Systematic Review and Meta-analysis
Source: JAMA Netw Open. 2023 Feb 10;6(2):e2256178. doi: 10.1001/jamanetworkopen.2022.56178 (PMC9918886; doi:10.1001/jamanetworkopen.2022.56178)
Supplement: Supplement 2. — Data Sharing Statement [file jamanetwopen-e2256178-s002.pdf]

Data Sharing Statement

Sperotto. Trends in In-Hospital Cardiac Arrest and Mortality Among Children With Cardiac Disease in the Intensive Care Unit. *JAMA Netw Open*. Published February 10, 2023. doi:10.1001/jamanetworkopen.2022.56178

Data
Data available: No

Additional Information
Explanation for why data not available: This manuscript does not include original data. Data are extracted from literature and are publicly available.
